# Comparative evaluation of pre-column derivatization strategies for PFAS analysis in paper-based food contact material using GC-MS

**DOI:** 10.1007/s00216-026-06683-z

**Published:** 2026-07-25

**Authors:** Charlotte Hauschildt, Christian Jung, Hajo Haase, Roland Becker

**Affiliations:** 1 Department of Analytical Chemistry and Reference Materials, Bundesanstalt für Materialforschung und -prüfung (BAM), Berlin, Germany; 2 Department of Food Chemistry and Toxicology, Technische Universität Berlin, Berlin, Germany

**Keywords:** PFAS, Gas chromatography-mass spectrometry, Derivatization, FCM, Automatization

## Abstract

The analysis of paper-based food contact materials (FCM) has gained increasing attention due to the widespread use of per- and polyfluoroalkyl substances (PFAS) and their known toxic effects and environmental persistence. As regulatory frameworks for PFAS in FCM remain limited, sensitive and reliable analytical methods are necessary to ensure food safety. While PFAS analysis is predominantly performed using liquid chromatography coupled to tandem mass spectrometry (LC-MS/MS), a systematic development of gas chromatography coupled to mass spectrometry (GC-MS)-based methods for multiple PFAS classes remains limited. In this study, two pre-column derivatization techniques were optimized and evaluated for GC-MS determination of perfluoroalkyl carboxylic acids (PFCA), fluorotelomer alcohols (FTOH), and fluorotelomer carboxylic acids (FTCA). Both derivatization methods enabled qualification and quantification of the targeted PFAS with comparable limits of detection in the low ng/mL range. N,N-Dimethylformamide dimethyl acetal (DMFDMA) derivatization proved to be more sensitive with a limit of detection down to 0.85 ng/mL. In addition, this derivatization approach was successfully automated within the GC-MS workflow, resulting in sample preparation times comparable to those of LC-MS-based methods. The application of this optimized method to paper-based FCM demonstrated its suitability for real world FCM analysis. Results of PFAS content per targeted PFAS of 26.6 to 261.4 ng/g highlight the potential of GC-MS analysis, particularly when combined with automated derivatization, as a viable alternative or complement to LC-MS for PFAS analysis in FCM.

**Graphical abstract:**

**Figure:**
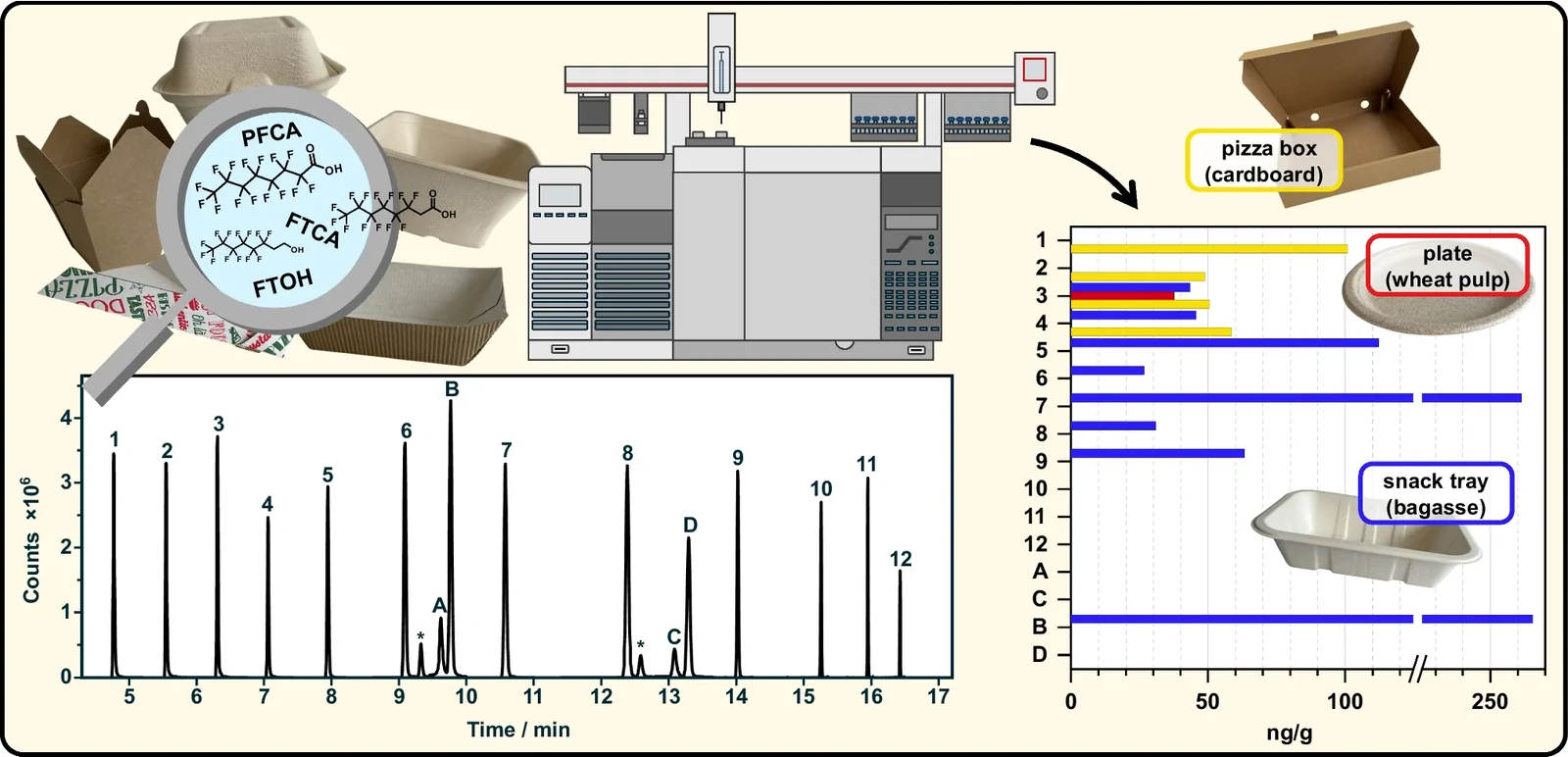

**Supplementary Information:**

The online version contains supplementary material available at https://doi.org/10.1007/s00216-026-06683-z.

## Introduction

Per- and polyfluoroalkyl substances (PFAS), commonly known as “forever chemicals”, have been in widespread industrial use since the 1940s [1–4], when polytetrafluoroethylene (PTFE) was first commercialized as Teflon® [5, 6]. In the following decades, the applications of PFAS expanded rapidly across the cosmetic, textile, electronics, and automotive industries, among others [7–9], due to their hydrophobic and oleophobic properties [1, 3, 8]. According to the *Organisation for Economic Co-operation and Development* (OECD), all compounds containing a fluorinated methyl (-CF_3_) or methylene (-CF_2_-) group are classified as PFAS, resulting in a variety of unique physicochemical properties [2, 10]. A total of 4730 PFAS can be assigned a CAS number recording to the OECD definition, while the overall group is estimated to comprise up to 10,000 different compounds [10, 11]. PFAS are nowadays associated with environmental persistence [12, 13], bioaccumulation [9, 14], adverse health effects [14–16], and toxicological risks [17, 18]. The first records of suspected toxic effects of PFAS were already made in the 1950 s [19, 20]. According to current knowledge, the assumed toxic effects range from carcinogenicity to neurotoxicity and systemic toxicity [14–17]. As a result of their toxic properties and ubiquitous occurrence [8, 11], some PFAS compounds have already been regulated. The regulatory groundwork for the EU was first laid by the REACH Regulation (EC No. 1907/2006) in 2006, which provided the EU’s initial comprehensive framework for controlling chemical substances of industrial relevance, including PFAS [21]. Other EU regulations include the ban of perfluorooctanesulfonic acid (PFOS) since December 2006 [22], and perfluorooctanoic acid (PFOA) since July 2020 [23]. As a representative example of numerous regulatory actions, the EU has set maximum levels for selected PFAS in certain foods of animal origin, including meat, fish, crustaceans, and eggs, since December 2022 under Commission Regulation (EU) 2022/2388 [24].

Food contact materials (FCM) are one of the pathways for human exposure to PFAS [25–28]. Direct contact with various food matrices, particularly at elevated temperatures, increases the migration of PFAS from FCM into food. Paper-based FCM are treated with PFAS to make them water- and oil-repellent, enabling their use as FCM [28–31]. PFAS have been detected in various paper-based FCM such as popcorn bags, baking paper, wrapping paper, burger boxes, and many more over the past decades [27, 32–36]. In addition, an increasing shift toward sustainability and the resulting transition from plastic to disposable and recyclable paper products has led to a significant increase in the consumption of paper-based FCM, making their analysis highly relevant to food safety [36–38]. To ensure food safety and compliance with these set limits in foodstuff, the development of analytical methods for the quantification of PFAS has been in focus for years. The current analytical standard for PFAS analysis is liquid chromatography coupled with (tandem) mass spectrometry (LC-MS/MS), which covers a broad spectrum of ionic PFAS such as perfluorocarboxylic acids (PFCA), perfluorosulfonic acid (PFSA), perfluoroalkyl phosphonic acid (PFPA), and nonionic PFAS with limit of detection (LOD) < 1 ppb [39–42]. Gas chromatography coupled with mass spectrometry (GC-MS) provides a less complex and more cost-efficient alternative [43, 44] that is well-suited for the analysis of nonionic PFAS such as FTOH and polyfluorinated sulfonamides (FOSA) [45–47]. A frequently cited limitation of GC-MS methods for PFAS detection is the requirement for derivatization, which is a time-consuming additional step during sample preparation [48–50]. For example, several derivatization approaches have been reported for the free carboxyl group of PFCA, including methylation [51–54], alkylation [13, 55, 56], silylation [50, 57], and amidation [43]. A further approach transformed PFCA into bis-(dimethylamino)-derivatives using N,N-dimethylformamide dimethyl acetal (DMFDMA) [58–60]. All these procedures have been applied to PFCA quantification in water [61, 62], soil [45, 52], foodstuff including milk and seafood, and food packaging [33, 60, 63]. In any case, GC-MS requires a quick and complete transformation into a derivative displaying improved chromatographic performance and analytical sensitivity.

Based on these approaches, this study optimized and compared two pre-column derivatization reagents for the simultaneous analysis of three PFAS classes, thereby investigating gas chromatography coupled to simple single quad mass spectrometry as a possibility for extending the analytical scope for these compounds [43, 48, 55, 60]. These methods enable the simultaneous determination of multiple PFCA, FTOH, and fluorotelomer carboxylic acids (FTCA), using the derivatization reagents DMFDMA and 2,3,4,5,6-pentafluorbenzylbromide (PFBBr). For the first time, a fully automated system for improved derivatization with DMFDMA has been developed. This approach integrates the derivatization step directly into the analysis, providing a fast, simple, and reproducible method. Following systematic optimization and comparative evaluation, the fully automated DMFDMA derivatization was implemented for the analysis of PFAS in paper-based FCM.

## Materials and methods

### Chemicals and standards

N,N-Dimethylformamide dimethyl acetal (DMFDMA, CAS 4637-24-5, > 98%) was purchased from Carl Roth GmbH + Co. KG (Karlsruhe, Germany). 2,3,4,5,6-pentafluorbenzylbromide (PFBBr, CAS 1765-40-8, 99%) was obtained from Merck/Sigma Aldrich (Darmstadt, Germany). All solvents used were of p.a. grade or SupraSolv® grade. Methanol (MeOH), acetonitrile (ACN), toluene, and acetone were purchased from CHEMSOLUTE, Th. Geyer (Renningen, Germany). Tert-butyl methyl ether (MTBE) (for gas chromatography, > 99.8%, SupraSolv®) was obtained from Merck. Potassium carbonate anhydrous (CAS 584-08-7) was supplied by J.T. Baker (Deventer, Netherlands) and sodium carbonate anhydrous (p.a., 99.8%, CAS 497-19-8) was purchased from CHEMSOLUTE, Th. Geyer. Ultrapure water (> 18.0 MΩ/cm at 25 °C) was produced with a Quantum® TEX Milli-Q from Merck. Further information regarding native and labelled standards used for GC-MS, as well as their abbreviations, can be found in the supplementary information (SI) Table S1.

### Stock solutions

A standard stock solution of 16 different PFAS, containing PFCA (C4-C12, C14, C16, C18), FTOH (6:2, 8:2), and FTCA (8:2, 10:2) (details in Table S1), was prepared at a concentration of 50 µg/mL in respective solvent depending on the derivatization method. Subsequently, stock solutions were diluted to the appropriate concentrations for analytical measurements. For method validation a concentration range of 10 to 200 ng/mL in the respective solvent was used. To determine extraction recoveries a concentration of 25 µg/mL in acetonitrile was used.

### Sample preparation

A total of eight paper-based FCM were collected for analysis. The materials were categorized based on their material properties: cardboard: *n* = 4, bagasse: *n* = 3, wheat pulp: *n* = 1 (details in Fig. 5). The materials were provided in the framework of the EU project Screenfood (23IND13, ScreenFood). For validation purposes, spiked FCM analysis was performed using an optimized extraction method. Material spiking was carried out by even applying PFAS stock solution at a concentration of 25 µg/mL in acetonitrile to the selected FCM. Following the evaporation of acetonitrile, the dry spiked FCM was cut into pieces measuring 1 cm^2^ with cleaned scissors rinsed with acetone and transferred to 15 mL PP Falcon™ Conical Centrifuge Tube (Thermo Fisher Scientific, Darmstadt, Germany). For material extraction, the respective raw material was cut into pieces of 1 cm^2^ with cleaned scissors rinsed with acetone without spiking. Per extraction 1 g of raw material was used. PFAS extraction was done by sonication at room temperature with 5 mL of ACN for a total duration of 15 min. Then the extract was collected and filtered through disposable 0.45 µm regenerated cellulose syringe filters (CHROMAFIL Xtra RC, 0.45 µm, Macherey–Nagel, Düren, Germany) to remove any fine particles. The extraction was repeated in three consecutive extraction cycles (EC). To 200 µL aliquots of each EC, 10 µL of internal standard (IS) mix at a concentration of 500 ng/mL in acetonitrile was added after the extraction.

### Derivatization with N,N-dimethylformamide dimethyl acetal

Derivatization was performed under optimized conditions by adding 10 µL of undiluted DMFDMA to a 200 µL aliquot of standard solution or acetonitrile sample extract. To ensure comparability, an automated sample preparation procedure was established using the autosampler MPS XL system (Gerstel, Mülheim a.d. Ruhr, Germany). In this procedure, 10 µL (2 × 5 µL) of DMFDMA was automatically added to a 200 µL aliquot, which was stored at 10 °C in the GC cooler tray. Following a 3 min incubation period with shaking at 300 rpm in the agitator module, the sample was injected into the cold injection system (CIS) of the GC. To program the automation script, the Gerstel Maestro1 software (version 3.5, Gerstel) was used.

### Derivatization with 2,3,4,5,6-pentafluorobenzylbromide

For the derivatization of PFAS, 12.5 µL of undiluted PFBBr and 10 µL of freshly prepared 1 M K_2_CO_3_ in ultrapure H_2_O were added to 1 mL aliquots of standard solution or acetone sample extract. The mixture was incubated at 65 °C for 60 min with gentle shaking at 400 rpm using the MHR 13 heated thermoshaker (HLC BioTech, Bovenden, Germany). Following incubation, the sample was cooled down to room temperature and subsequently filtered through a disposable 0.45 µm regenerated cellulose syringe filter. The sample was then automatically injected into the CIS of the GC-system for further analysis. All experimental parameters were optimized prior to analysis.

### Instrumental analysis

GC-MS measurements were performed using a 7890 A gas chromatograph (Agilent Technologies, Waldbronn, Germany) equipped with a Gerstel Maestro autosampler (version 3.5, Gerstel, Mülheim a.d. Ruhr, Germany), a CIS (Gerstel), and a 5975 C linear quadrupole mass spectrometer (Agilent Technologies). The measurement system was operated using Mass Hunter Workstation software (version 10.0.368, Agilent Technologies) and integrated Maestro software (version 3.5, Gerstel) for automatic injections. The CIS program started with an equilibration time of 0.1 min at 30 °C and then a gradient of 12 °C/s to 275 °C, held for 5 min. In the injector, a 2 mm deactivated multiple-baffle liner was used. Injections were performed in split mode (1:5 or 1:2) or splitless mode, depending on the measurement method. The injected volume of all samples was 1 µL. The carrier gas was helium (5.0, Linde, Pullach, Germany) with a constant flow rate of 1.0 mL/min. Separation was achieved on a HP-5 MS capillary column (30 m × 0.25 mm id., 0.25 µm film thickness; Agilent J&W Scientific).

The oven program for the DMFDMA derivatives started at 40 °C (held for 3 min) and was ramped to 92.5 °C (held for 3 min) with 15 °C/min, then to 100 °C with 2 °C/min and finally to 320 °C with 35 °C/min (held for 3 min). The total duration of this optimized oven program is 22.54 min. The oven program for the PFBBr derivatives started at 40 °C (held for 1 min) and was ramped to 70 °C with 10 °C/min, followed by 30 °C/min to 95 °C (held for 1 min) and then to 100 °C with 5 °C/min. In the final steps, the temperature was ramped to 225 °C with 15 °C/min and then to 300 °C with a 30 °C/min (held for 2 min). The total duration of this optimized oven program is 19.66 min. The transfer line temperature was set to 320 °C for DMFDMA measurements and to 300 °C for PFBBr measurements. The mass spectrometer was operated in electron impact ionization (EI) mode at 70 eV, and the ion source was maintained at 230 °C. Mass spectra of the derivatized samples and extracts were acquired in full scan mode over a m/z range of 35 to 1000 for method development or in selected ion monitoring (SIM) mode for quantification. The data were qualitatively analyzed using *Mass Hunter Workstation Qualitative Analysis* software (version 10.0, Agilent Technologies) and quantitatively analyzed using *Mass Hunter Workstation Quantitative Analysis for GCMS and LCMS* software (version 10.1, Agilent Technologies). For method calibration, eight equidistant calibration points in the range of 10 to 200 ng/mL per PFAS compound were measured in triplicates.

### Quality assurance/quality control

PFAS-free consumables (PFAS-free septa, polypropylene GC vials, and septum-free syringes for automated injection) were used in all experiments. All solvents were tested for PFAS content (solvent blanks). Solvent blanks (*n* = 3) were determined using 200 mL solvent spiked with 25 ng of the labelled IS mixture (10 μL, concentration of 10 ng/mL in ACN). To ensure reproducibility and rule out system contamination, method and system blanks were measured for each analysis. Method blanks (*n* = 3) were performed injecting ACN after sample treatment. Spiked material experiments were conducted to ensure the quality of real sample analysis. To ensure repeatability, all spike experiments were conducted in triplicates. First, the number of extraction steps required for quantitative recovery of the analytes was tested. For this purpose, all analytes were spiked in a high concentration (1 µg/mL) onto selected FCM (details in the “Sample preparation” section). The limit of detection (LOD) and limit of quantification (LOQ) were obtained using the calibration curve method based on German standard DIN 32645:2008 [64] with the calibration points described in the proceeding section (*n* = 3). Details on this DIN method can be found in the SI.

## Results and discussion

### Derivatization of PFAS with N,N-dimethylformamide dimethyl acetal

**Fig. 1:**
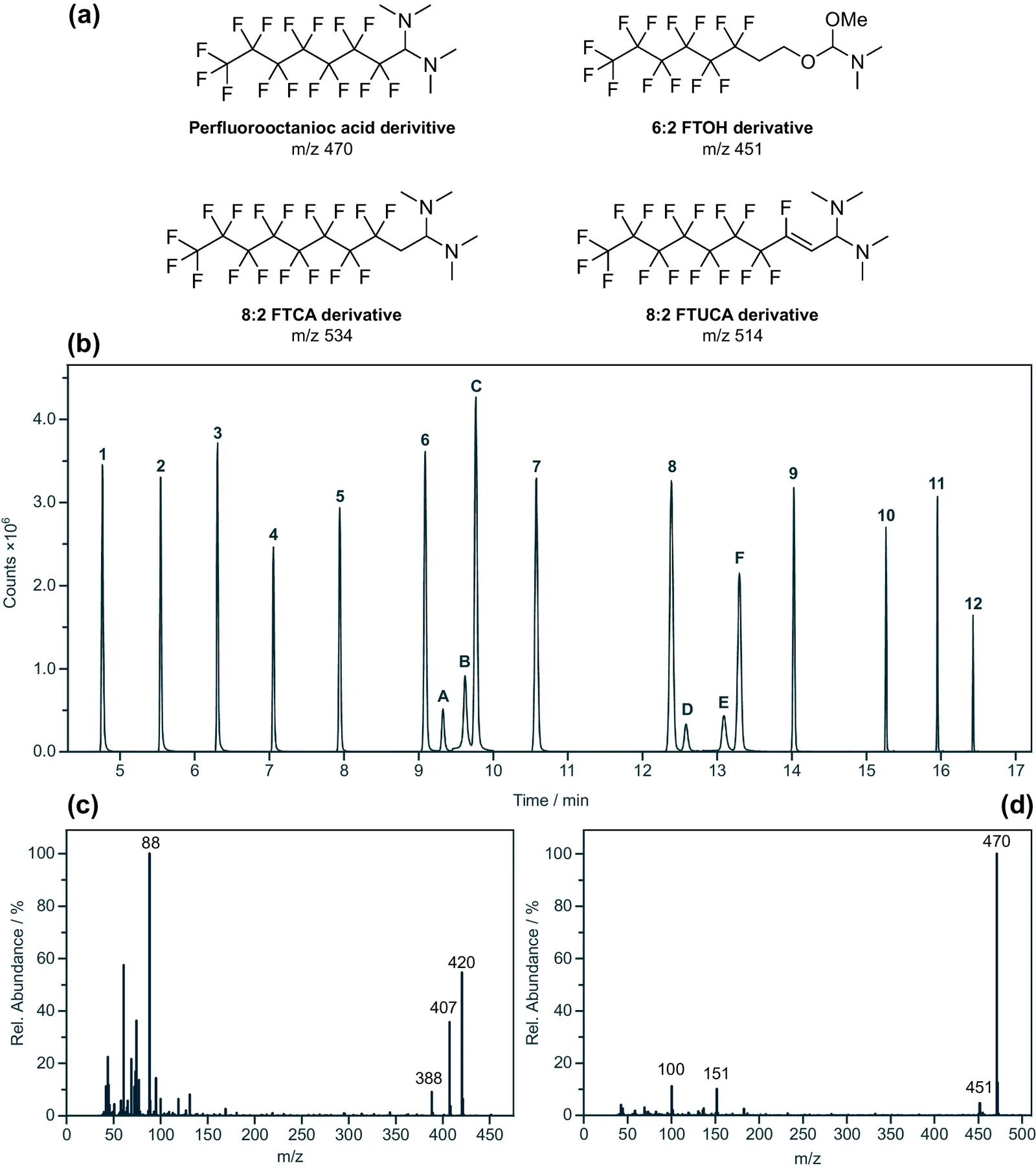
** a** Structure of DMFDMA-derivatized, unfragmented molecules (molecular peaks) of PFOA, 6:2 FTOH, 8:2 FTCA, 8:2 FTUCA; **b** selected ion monitoring chromatogram of a gas chromatographic (GC) separation of 16 PFAS (1–12: PFCA C4-C12/C14/C16/C18; A + D: FTUCA (8:2, 10:2), B + E: FTOH (6:2, 8:2), C + F: FTCA (8:2, 10:2)) after derivatization with DMFDMA; **c** mass spectrum (EI, 70 eV) of 6:2 FTOH derivative; **d** mass spectrum (EI, 70 eV) of 8:2 FTCA derivative

DMFDMA has particularly been used for the derivatization of carboxylic acids [58–60]. The reaction with the carboxyl groups of PFCA yields bis-(dimethylamino)-derivatives (see Fig. 1a), which are more volatile and thermally stable, facilitating their efficient separation and detection by GC-MS [58, 60]. For the first time, this approach was extended to further PFAS classes to convert these into DMFDMA derivatives, enabling analysis of PFCA, FTOH, and FTCA simultaneously. The unsaturated fluorotelomer carboxylic acid (FTUCA) was also qualified using this method. FTUCA differs from FTCA in terms of HF-loss and has been reported as a possible intermediate degradation product [65–67]. The presence of FTUCA as a by-product in the FTCA standard was confirmed by LC-MS/MS analysis. The derivatization was performed straight forward by adding undiluted DMFDMA, followed by direct injection onto the CIS of the GC-MS system. The PFAS derivatives could be completely separated in less than 17 min under optimized GC-MS conditions (see Fig. 1b). To further simplify and standardize sample preparation, an automated sample preparation/derivatization was implemented using the Gerstel MPS XL Twister system. This step eliminates the need for manual derivatization, reducing the effort required for sample preparation. The automation results in a reduction of time required for sample preparation and an increase in reproducibility. To ensure reliable automated derivatization and thorough homogenization of the sample with the derivatization reagent, an incubation step with gentle shaking is integrated prior to injection.

The mass spectrum of derivatized 6:2 FTOH (see. Fig. 1c) shows fragment ions with m/z of 88, 388, 407, and 420 with high intensity. The base peak of m/z 88 corresponds to the molecular peak losing a methoxy group [M-CH_3_O]^+^*.* The highest fragment at m/z 420 corresponds to [C_11_H_11_F_13_NO]^+^. This fragment breaks further into the smaller fragment of m/z 407 [C_10_H_10_F_13_NO] losing a methyl group. Further fragmentation to m/z 388 with a mass difference of 19 indicates the loss of a fluorine atom. In case of derivatized 8:2 FTCA m/z 470 is the most abundant fragment (see Fig. 1d). This fragment results from the molecular ion losing a dimethylamino group [M-N(CH_3_)_2_]⁺. The further fragmentation to m/z 451 indicates the loss of fluorine atom. The fragmentation pathway of 8:2 FTUCA is equivalent to that of 8:2 FTCA because both structures differ only in terms of HF loss (difference of 20 m/z). The fragmentation pathway of derivatized PFOA, exemplary for PFCA, 6:2 FTOH, and 8:2 FTCA were depicted in Fig. S1 in the SI. Representative mass spectra of PFOA, 6:2 FTOH, 8:2 FTCA, and 8:2 FTUCA can be found in Fig. S2. 8:2 and 10:2 FTUCA, which were detected as by-products of the FTCA standards, are not optimized or quantified further. However, the results demonstrate that FTUCA can be qualified using the described method, making it feasible to integrate this PFAS class into the analysis.

### Method optimization

**Fig. 2:**
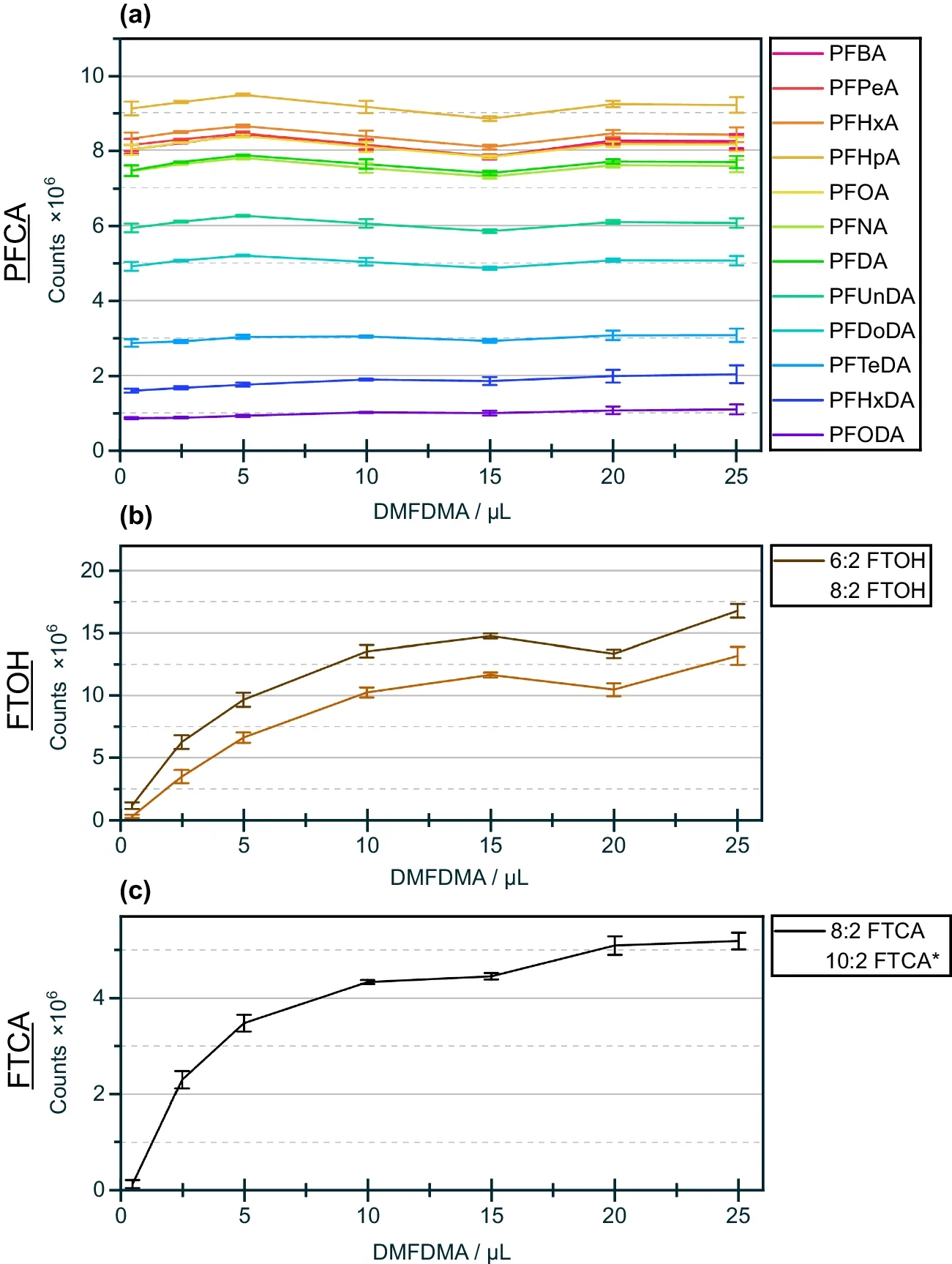
Optimization of the DMFDMA volume for the derivatization of PFCA (**a**), FTOH (**b**), and FTCA (**c**). Evaluation of different volumes of DMFDMA (0.5–25 µL) in relation to a 200 µL aliquot of PFAS standard mix in acetonitrile, *10:2 FTCA was not analyzed, means and standard deviation (*n* = 3)

The derivatization conditions using DMFDMA were systematically optimized to ensure reliable and simultaneous detection of PFCA, FTOH, and FTCA. The first parameter investigated was the volume of the DMFDMA used for derivatization. The results are summarized for each PFAS class (see Fig. 2). To evaluate the effect of reagent volume on the signal intensity, reagent volumes ranging from 0.5 to 25 µL were tested. The resulting chromatographic peak areas were used to evaluate performance. For PFCA derivatives, the signal intensity was largely unaffected by the volume of DMFDMA (see Fig. 2a). In contrast, the signal intensity for derivatized FTOH depended strongly on the volume of DMFDMA within the range of 0.5–10 µL; less significant effects were observed at volumes between 10 and 25 µL. The derivatized 8:2 FTCA exhibited a saturating exponential curve that reached its plateau at approximately 10 µL of derivatization reagent (see Fig. 2c). As a result, a concentration of 10 µL DMFDMA was used for all further experiments.

Additionally, the influence of various solvents on derivatization efficiency was evaluated. The purpose of this optimization was to identify suitable solvents for subsequent extractions of PFAS-containing FCM. For this purpose, common solvents (MTBE, acetonitrile, ethyl acetate, acetone, and methanol) were compared with each other. Details are presented in Fig. S3. The results show that all solvents except methanol are suitable for the dilution of PFAS standards prior to DMFDMA derivatization. Methanol causes a rapid decrease in signal intensity within short time, resulting in inconsistent and unreliable measurements. For the analysis of FTOH neither ethyl acetate nor methanol led to sufficient derivatization. However, derivatization with DMFDMA works with MTBE, ACN, and acetone for all three PFAS classes. Furthermore, the stability of the derivatized PFAS was analyzed over a 24 h period. To assess this, the samples were stored at 15 °C in a GC cooler tray and measured hourly. As shown in Fig. S4, PFCA derivatives of DMFDMA remained largely stable with a slight decrease over the 24 h period. In contrast, FTOH derivatives are unstable over time. Intensity drops significantly over the 24 h period. Although derivatization for FTOH appears to work, the conditions would need to be optimized further to form stable products. The signal intensity of derivatized 8:2 FTCA increased slightly over the 24 h period. To enable reproducible quantification of FTOH despite the limited stability of the DMFDMA-derived product, an automated procedure with consistent timing throughout the entire sequence was developed (details of this automatization in the “Materials and methods” section).

### Quantification

Following a successful optimization of the derivatization and chromatographic separation conditions, the LOD and LOQ as well as the coefficients of determination (*R*^2^) were determined for each PFAS (see SI **DIN 32645:2008**). The LOD and LOQ were calculated using linear regression from eight measurement points in the range of 10–200 ng/mL (*n* = 3). Table 1 summarizes the chromatographic parameters, detection and quantification limits, and coefficient of determination that were used to validate the method and quantify PFAS in FCM.

**Table 1:** GC-MS parameter for all PFAS target analytes: retention time (RT), quantitative and qualitative ions, limit of detection (LOD) and limit of quantification (LOQ), and the coefficient of determination (*R*^2^) for each target analyte after DMFDMA derivatization

PFAS	RT (min)	Quantitative ion (m/z)	Qualitative ions (m/z)	*R*^2^	LOD (ng/mL)	LOQ (ng/mL)
PFBA	4.78	226	176, 107	0.9995	1.43	5.29
PFPeA	5.55	276	257, 226	0.9999	1.02	3.80
PFHxA	6.31	326	307, 276	0.9999	1.16	4.32
PFHpA	7.06	376	357, 326	0.9999	1.36	5.04
PFOA	7.94	426	407, 376	0.9999	1.20	4.46
PFNA	9.08	476	457, 426	0.9999	0.85	3.17
PFDA	10.57	526	507, 476	0.9999	1.08	4.04
PFUnDA	12.37	576	557, 526	0.9999	1.22	4.55
PFDoDA	14.01	626	607, 576	0.9988	4.49	15.85
PFTeDA	15.26	726	707, 676	0.9983	5.41	18.84
PFHxDA	15.94	826	807, 776	0.9984	5.37	18.70
PFODA	16.42	926	907, 876	0.9989	3.52	12.42
6:2 FTOH	9.62	420	407, 388	0.9994	4.87	17.51
8:2 FTOH	13.10	520	507, 488	0.9989	6.94	24.16
8:2 FTCA	9.77	470	451, 151	0.9982	9.07	30.65
10:2 FTCA	13.28	570	551, 151	0.9989	9.19	31.26

The optimized method exhibited good linearity for all target analytes, with calibration ranges spanning from 10 to 200 ng/mL with *R*^2^ ranging from 0.9982 to 0.9999. These *R*^2^ values exceeding 0.9982 demonstrate that the linear regression model satisfactorily explains the observed data across all targeted analytes. The LODs for the PFCA derivatives ranged from 0.85 to 5.41 ng/mL, whereas LODs for FTOH and FTCA were between 6.94 and 9.19 ng/mL. Compared with literature data reporting PFAS concentrations in paper-based food FCM, the achieved LODs are within the concentration ranges previously reported [28, 68–70]. Therefore, the method presented here establishes a solid foundation for PFAS analysis in FCM. Compared to other FCM studies conducted using LC-MS/MS with LODs ranging down to 0.04 ng/g, subsequent refinements may further enhance its sensitivity [70, 71]. These improvements in sensitivity may be attained, for example by increasing quantification performance, e.g., by GC-MS/MS application for more sensitive trace-level analysis.

### Derivatization of PFAS with 2,3,4,5,6-pentafluorbenzylbromide

**Fig. 3:**
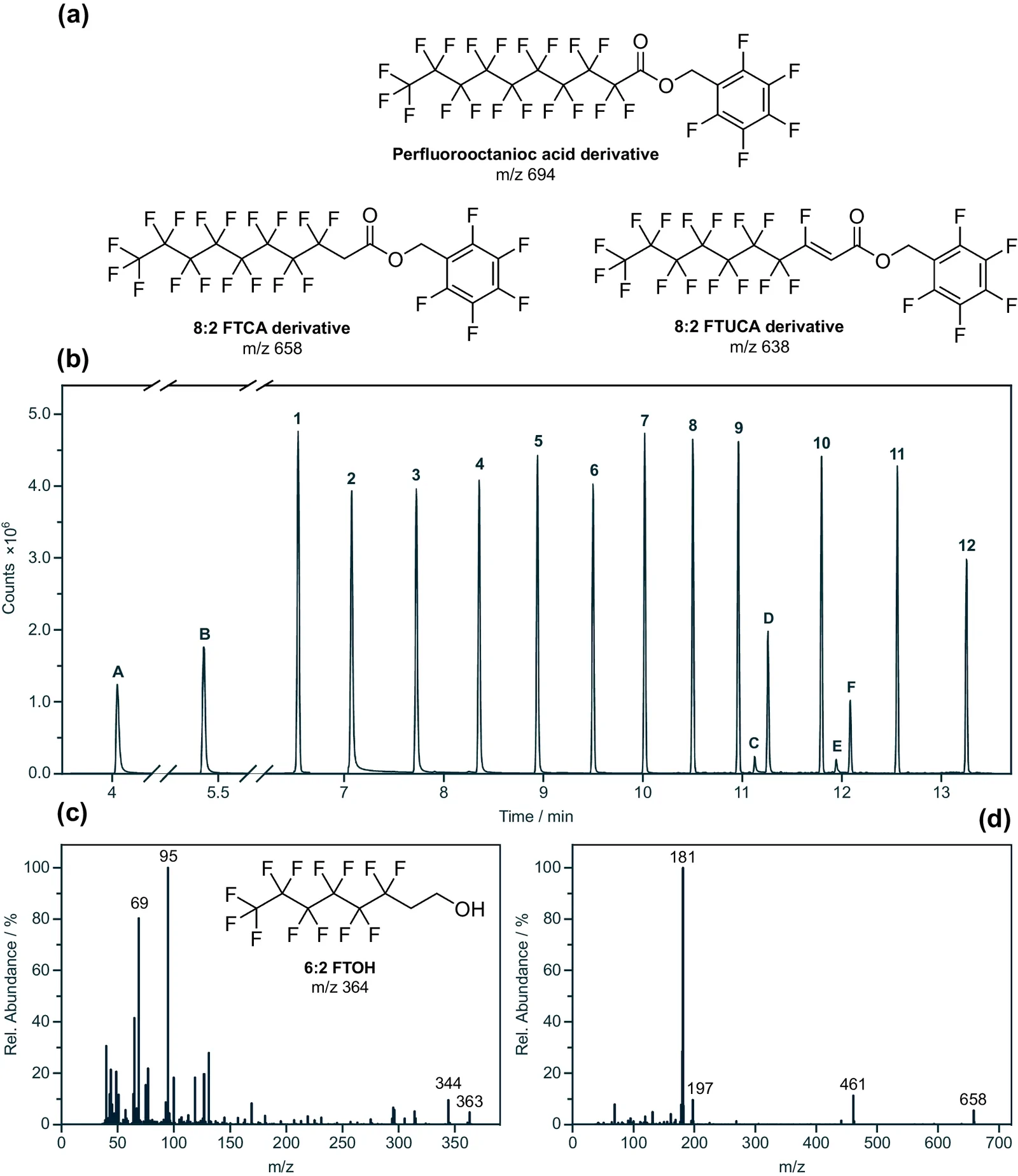
** a** Structure of PFBBr-derivatized, unfragmented PFAS molecules (molecular peaks); **b** selected ion monitoring (SIM) chromatogram of a gas chromatographic (GC) separation of 16 PFAS (1–12: PFCA C4-C12/C14/C16/C18; A + B: 6:2 and 8:2 FTOH, C + E: 8:2 and 10:2 FTUCA, D + F: 8:2 and 10:2 FTCA) after derivatization with 2,3,4,5,6-pentafluorbenzylbromide (PFBBr); **c** mass spectrum (EI, 70 eV) of underivatized 6:2 FTOH; **d** mass spectrum (EI, 70 eV) of 8:2 FTCA derivative

PFAS derivatization with PFBBr was performed under optimized conditions by adding the undiluted derivatization reagent and 1 M K_2_CO_3_ to the sample aliquot, followed by an incubation step at 65 °C for 60 min. Although the resulting pentafluorobenzyl ester exhibit higher molecular weights (see Fig. 3a), they remain suitable for GC analysis due to their relatively low boiling points and high volatility [56]. Under optimized GC conditions, the separation of FTOH as well as PFCA, FTCA, and FTUCA derivatives was achieved in less than 14 min (see Fig. 3b).

Figure 3c and d show the representative spectra of 6:2 FTOH and 8:2 FTCA after PFBBr treatment, indicating that only FTCA is derivatized using PFBBr. The mass spectrum FTOH shows fragmentation ions of its non-derivatized form, which has a molecular mass of 364.10 g/mol. The most abundant peak of m/z 363 correlates to [M-H]^+^. It is known from literature that the characteristic peaks of 6:2 FTOH are m/z 95 [C_3_H_5_F_2_O]^+^ as its base peak and m/z 69 [CF_3_]^+^ as the second highest [72]. The characteristic fragment of m/z 181, which corresponds to the pentafluorobenzylic cation, is not present after PFBBr treatment. That proves that no derivatization of FTOH with PFBBr has occurred. In contrast, the base peak of derivatized 8:2 FTCA is m/z 181 (see Fig. 3d). The fragment of m/z 658 results from the molecular ion losing the fluorinated benzyl alcohol group. The fragmentation pathway of 8:2 FTUCA is equivalent to that of 8:2 FTCA because both PFAS differ only in terms of HF loss (a difference of 20 m/z). The fragmentation pathways of derivatized PFOA, exemplary for PFCA, 6:2 FTOH, and 8:2 FTCA, were depicted Fig. S5 in the SI. Representative mass spectra of PFOA, 6:2 FTOH, 8:2 FTCA, and 8:2 FTUCA can be found in Fig. S6. FTUCA, which was detected as by-product of the FTCA standard, is not optimized or quantified further. However, the results demonstrate that FTUCA can be qualified using the described method, making it feasible to integrate this PFAS class into the analysis.

### Method optimization

**Fig. 4:**
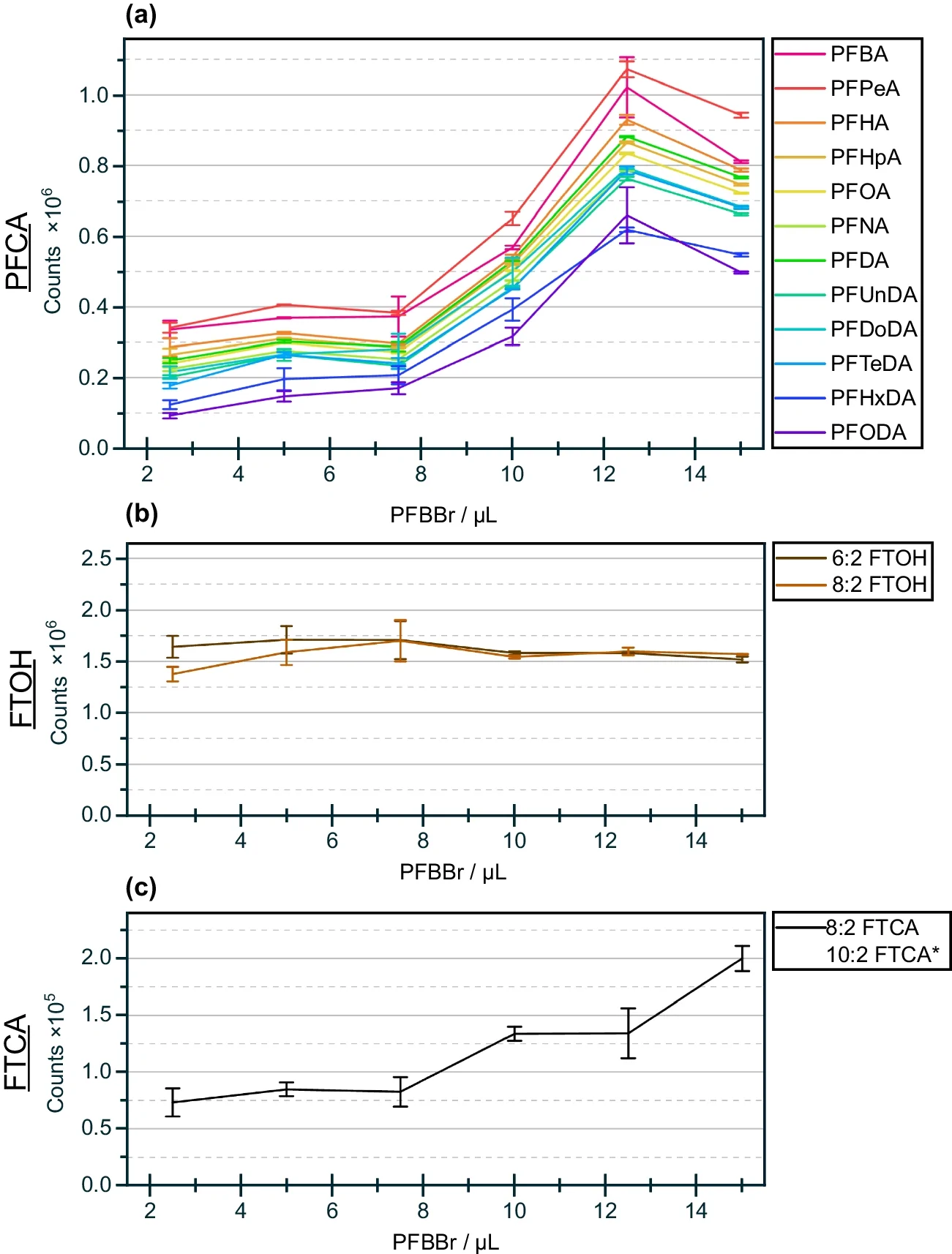
Optimization of the derivatization reagent volume of PFBBr for PFCA (**a**), FTOH (**b**), and FTCA (**c**) analysis. Evaluation of different volumes (2*.*5–15 µL) of PFBBr in relation to a 1 mL sample of PFAS standard mix. *10:2 FTCA was not analyzed, means and standard deviation (*n* = 3)

Derivatization with PFBBr involves multiple parameters that can influence derivatization efficiency. To determine optimal conditions, these parameters were systematically optimized for PFCA, FTOH, and FTCA. Specifically, the effects of the amount of PFBBr, the choice and amount of carbonate used, the reaction temperature, and the incubation time were investigated. The results for the variation of PFBBr volume were summarized for each PFAS class (see Fig. 4). To assess the impact of PFBBr on the derivatization efficiency of PFCA, FTOH, and FTCA, the volume was varied from 2.5 to 15 µL. Derivatization efficiency was evaluated based on the chromatographic peak areas of the derivatized PFAS. For PFCA, the amount of PFBBr had a significant effect on derivatization efficiency. The optimal volume was found to be 12.5 µL of PFBBr (see Fig. 4a). In contrast, the experimental data show that the FTOH response does not depend on the volume of applied PFBBr in the range of 2.5–15 µL. This further confirms that FTOH does not react with PFBBr and is not derivatized under the conditions applied. For derivatized 8:2 FTCA higher signal intensities were observed at increased amounts of PFBBr. Consequently, 12.5 µL of PFBBr was used for all subsequent experiments.

In addition to the volume of PFBBr, further experimental parameters of the derivatization method were examined and optimized. These included the incubation temperature and duration, the type of added carbonate and its volume. The results can be seen in Fig. S7. These show that the incubation time between 30 and 90 min has almost no effect on the reaction. The incubation temperature, on the other hand, does affect the reaction. The signal intensity increases with temperature and asymptotically approaches a saturation level at elevated temperatures. Higher temperatures than 75 °C were not considered due to the solvent's vapor pressure. Using either Na_2_CO_3_ or K_2_CO_3_ leads to comparable results for all PFAS classes. Increasing the volume of K_2_CO_3_ solution slightly increased the signal intensity of derivatized PFCA and FTCA. A saturation level was reached at 10 µL added to a 1 mL aliquot. Therefore, the reaction parameters were set to 10 µL of 1 M K_2_CO_3_, 1 h incubation time, and 65 °C as the incubation temperature for all subsequent experiments. In addition, the stability of the PFBBr-derivatized PFAS was investigated. Therefore, each sample was analyzed at hourly intervals over a 24 h period. As shown in Fig. S8, PFCA and FTCA derivatives, as well as FTOH, remained largely stable throughout the 24 h observation period.

### Quantification

After optimizing the derivatization parameters and the chromatographic separation conditions, the LOD, LOQ, and *R*^2^ were evaluated for each PFAS compound using the calibration curve method mentioned above. Details can be found in SI. Table 2 shows each retention time (RT), quantitative and qualitative ions used, and the LOD, LOQ, and coefficient of determination.

**Table 2:** GC-MS parameter for all PFAS target analytes: retention time (RT), quantitative and qualitative ions, limit of detection (LOD) and limit of quantification (LOQ), and the coefficient of determination (*R*^2^) according to each target analyte after PFBBr treatment

PFAS	RT (min)	Quantitative ion (m/z)	Qualitative ions (m/z)	*R*^2^	LOD (ng/mL)	LOQ (ng/mL)
PFBA	6.43	394	181, 131	0.9985	8.30	29.15
PFPeA	6.90	444	181, 131	0.9983	8.96	31.37
PFHxA	7.55	494	181, 131	0.9984	8.69	30.47
PFHpA	8.19	544	181, 131	0.9984	8.50	29.83
PFOA	8.78	181	594, 131	0.9993	5.78	20.64
PFNA	9.35	181	644, 131	0.9996	3.85	13.91
PFDA	9.87	181	694, 131	0.9990	6.58	23.35
PFUnDA	10.36	181	744, 131	0.9990	6.84	24.23
PFDoDA	10.81	181	794, 131	0.9987	7.64	26.95
PFTeDA	11.97	181	894, 131	0.9995	4.69	16.87
PFHxDA	12.42	181	994, 131	0.9994	4.93	17.68
PFODA	13.11	181	169*, 131	0.9995	4.72	16.96
6:2 FTOH	3.91	95	363, 344	0.9992	5.88	20.98
8:2 FTOH	5.26	95	463, 444	0.9993	5.49	19.62
8:2 FTCA	11.12	658	181, 461	0.9978	13.19	43.99
10:2 FTCA	11.95	758	181, 561	n.d	n.d	n.d

*m/z 1094 is out of detection range. *n.d.*: not determined

The optimized method for PFBBr derivatization exhibited good linearity for all target analytes, with *R*^2^ at least 0.9978 and calibration ranges spanning from 10 to 200 ng/mL. These *R*^2^ values exceeding 0.9978 demonstrate that the linear regression model satisfactorily explains the observed data across all targeted analytes. The LODs for the PFCA derivatives ranged from 3.85 to 8.96 ng/mL, whereas LODs for FTOH and FTCA were between 5.49 and 13.19 ng/mL. The detection limits achieved for PFAS analysis with PFBBr are also within the concentration ranges previously reported in the literature for PFAS concentrations in paper-based FCM [28, 68–70]. Both methods establish a solid foundation for PFAS analysis in FCM. The LOQs achieved are sufficient for the determination of the most frequently detected PFAS. While LC-MS/MS-based methods have demonstrated higher sensitivity, partly attributable to the use of MS/MS detection and display as of yet a wider range of detectable PFAS, GC-MS is capable of quantifying PFCAs and FTOHs as the compound classes most relevant in FCM. Further method development could expand this range.

### Derivatization method comparison

Both methods are suitable for analyzing PFCA, FTCA, and FTOH simultaneously in less than 20 min with comparable LOD ranges in the lower ng/mL range. Therefore, these methods largely meet the recently defined limit values of the EU regulation 2025/40 article 5(5), where limit values of 25 ppb for any PFAS as measured with targeted PFAS analysis has been defined for new FCM placed on the market [73]. A comparison of both methods shows that the main advantage of DMFDMA derivatization over PFBBr is its simplicity and time-efficiency as a one-step reaction without incubation time or temperature control. Automation further accelerates this process and ensures a high degree of reproducibility. Due to this automation, preparing samples using GC-MS is comparable in speed to analyzing PFAS using LC-MS. An advantage of derivatization using PFBBr is the stability of the resulting products and the fast chromatographic separation. Automatic sample preparation has not yet been developed because the addition of carbonate leads to precipitation and tends to cause clogging of the injection needle without prior filtration. However, improved technology could make automation achievable and greatly shorten the sample preparation process by interleaving the individual samples. Although, the DMFDMA method was preferred for further usage due to its easy implementation and automation, and the lower LODs between 0.8 and 9.7 ng/mL.

### Method application to paper-based food contact materials

**Fig. 5:**
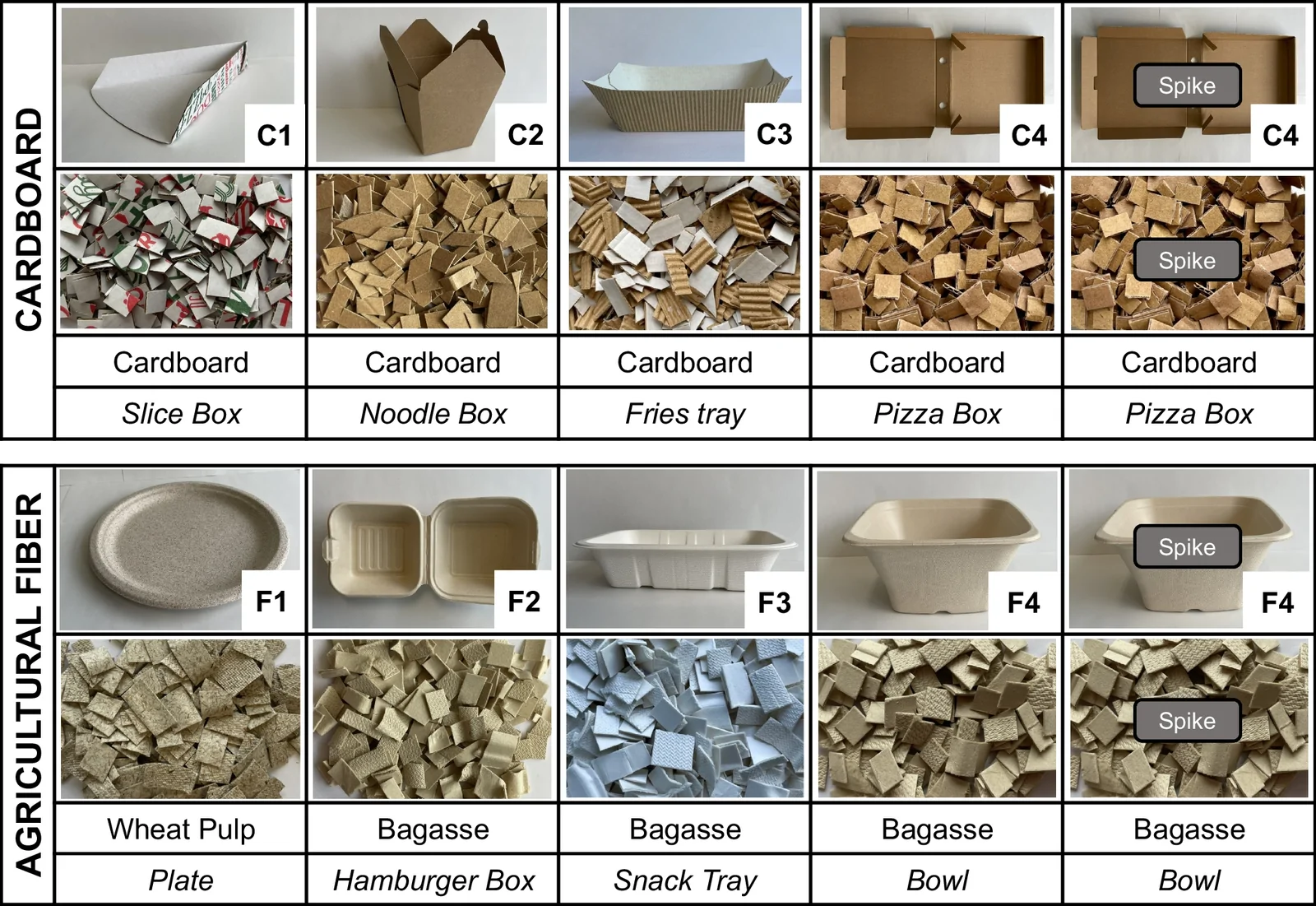
Overview of analyzed paper-based food contact materials with details on material composition and intended use. Categorized in cardboard materials (C1–C4) and agricultural fiber materials (F1–F4). One material of each category was used for spiked experiments to investigate extraction and recovery rates (C4, F4)

PFAS are widely used in paper-based FCM due to their water- and oil-repellent properties, which are highly relevant for food industry applications [30, 74]. Thus, paper-based FCM were tested for PFAS. For this purpose, various materials were selected (cardboard: *n* = 4, bagasse: *n* = 3, wheat pulp: *n* = 1). All materials were intended for direct contact with food at high temperatures. The optimized DMFDMA method described above was applied to analyze PFAS in eight FCM. Figure 5 shows the selected FCM made from cardboard, wheat pulp and bagasse divided into two categories of carboard materials and agricultural fiber materials.

To validate the developed DMFDMA derivatization method, spiked materials were initially tested. For this purpose, two different FCM, one of each category, were tested with a standard mix containing all three PFAS classes at 1 µg/mL. A labelled internal standard, added after the extraction, was used to compensate for instrument variability and drift effects. The extraction efficiency of each extraction cycle was tested in five consecutive steps (*n* = 3). The results show that the extraction efficiency in three consecutive steps of a total of 5 steps tested averages 94.5% ± 2.5% for cardboard material and 88.9% ± 1.7% for bagasse material. The low RSD values obtained from triplicate analysis indicate good precision of the method, confirming that the analytical procedure provides consistent and reliable results across repeated measurements. Details on the extraction efficiency of each cycle and its relative standard deviation (RSD) for each targeted PFAS are shown in Tables S2 and S3. As a result, three consecutive steps were subsequently determined to be sufficient for further experiments. In addition, the recovery of spiked PFAS standard mixture was determined in both material categories. The recovery rates for spiked cardboard samples extracted in three consecutive cycles range from 73.6 and 108.8% for PFCA, around 15% for FTOH, and between 21.3 and 42.3% for FTCA. For fiber materials, the recovery rates differ. For PFCA, the recovery in three consecutive cycles is between 26.6 and 73.1%, for FTOH between 130.9 and 144.2%, and for FTCA between 23.9 and 35.4% (details in Table S4). The overall precision of the recovery rates for the main extraction step (EC1) is very good, with RSD values below 10% for all analytes except 6:2 FTOH in the cardboard material with 14.3%, confirming the reliability and reproducibility of the extraction procedure. However, these results also indicate different material properties of each category, which may result from variations in manufacturing processes and PFAS application process used. This concept is further supported by the clearly distinguishable visual characteristics of the investigated FCM. Notably, the cardboard materials exhibit a markedly more porous surface structure, whereas the fiber-based materials appear considerably denser and, consequently, less absorbent. Furthermore, the food contact surfaces of all fiber-based materials appear significantly smoother, suggesting that they have undergone some form of surface treatment. In contrast, the surfaces of all cardboard materials are uniform in texture and appear to be untreated, except for partially printed areas. These observed differences in surface morphology may be attributable to the specific methods by which PFAS are incorporated into paper-based materials, such as coating, laminating, or sizing [30, 31, 75]. Therefore, further optimization of sample extraction specific to each material type may be required to make the analysis more effective.

This optimized method was applied to determine various PFAS in eight FCM made from cardboard and agricultural fibers. Three FCM containing PFCA and FTCA concentrations between 26.7 ng/g and 274.9 ng/g were detected (details in Tables S5 and S6). The highest amounts of PFAS were found in the snack tray (F3) made from bagasse. The predominant PFAS in the analyzed snack tray are 8:2 FTCA and PFDA. In addition, PFCA with a chain length of six to twelve could be detected. Also, in material C4 (pizza box made from cardboard) and F1 (plate made from wheat pulp), various PFCA were detected. In the analyzed pizza box, only elevated levels of short-chain PFCA between 31.1 and 62.6 ng/g were measured. FTOH were not detected in any of the analyzed materials, which could be due to the absence of FTOH in the selected materials. However, the very low recovery rates in cardboard and elevated recovery rates in fiber materials may also suggest the need for a further optimized extraction method. This could be likely related to the high volatility of FTOHs and the ubiquitous presence of FTOH in the air. In general, the PFAS concentrations determined in this study are consistent with values reported in recent literature [68, 76–79]. Most published data on PFAS analysis in FCM are based on LC-MS/MS methods. The GC-MS method presented here yields comparable results and demonstrates good reproducibility, as evidenced by low relative standard deviations, indicating its suitability as a complementary analytical approach. Furthermore, the limit values applicable from August 2026 for individual PFAS compounds, as well as the sum of PFAS measured by targeted PFAS analysis, as set out in EU Regulation 2025/40, are also largely met with the analysis presented [73]. From a green analytical chemistry perspective, the method presented here is characterized by low solvent consumption and comparably fast analysis times with sixteen analytes per run. The partly automated workflow further reduces the potential for human error and ensures ease of implementation in routine laboratory settings. Overall, this method offers a practical and resource-efficient alternative for the determination of PFAS in paper-based FCM.

## Conclusion

This study optimized and validated two pre-column derivatization methods for GC-MS enabling the simultaneous determination of sixteen legacy and emerging PFAS including PFCA, FTOH, and FTCA. These methods are also shown to be suitable for the qualification of FTUCA but were not optimized for quantification. Key parameters affecting derivatization efficiency were systematically optimized, and an automated derivatization procedure was implemented for the DMFDMA-based approach, achieving sample preparation times comparable to LC-MS-based methods. To the best of our knowledge, this is the first report of an automated derivatization strategy for PFAS analysis by GC-MS. Both approaches achieved detection limits in the lower ng/mL range, which proved to be sufficient for the reliable analysis of paper-based FCM exhibiting elevated contamination levels. The application to FCM analysis demonstrated the suitability of this method for the rapid and sensitive analysis with GC-MS, with elevated PFAS levels detected in both cardboard and agricultural fiber-based materials, consistent with previous reports. Further improvements, for example coupling the developed derivatization methods with GC-MS/MS or expanding the PFAS range could enhance analytical sensitivity and selectivity even further. With continued optimization, this method may complement, and in selected applications provide an alternative to established LC-MS-based methods.

## Supplementary Information

Below is the link to the electronic supplementary material.

Supplementary file1 (PDF 1.20 MB)

## Data Availability

Data will be made available on request.
