# Supplementary material for: Comparative evaluation of pre-column derivatization strategies for PFAS analysis in paper-based food contact material using GC-MS
Source: Anal Bioanal Chem. 2026 Jul 25;418(21):6861–75. doi: 10.1007/s00216-026-06683-z (PMC13658404; doi:10.1007/s00216-026-06683-z)
Supplement: Supplementary file 1 [file 216_2026_6683_MOESM1_ESM.pdf]

Supplementary Information (SI)

Comparative evaluation of pre-column derivatization strategies for PFAS analysis in paper-based food contact material using GC-MS

In: Analytical and Bioanalytical Chemistry (ABC) - Springer Nature

Charlotte Hauschildt [1,2], Christian Jung [1], Hajo Haase [2], Roland Becker [1,*]

[1] Bundesanstalt für Materialforschung und -prüfung (BAM), Department of Analytical Chemistry and Reference Materials, Berlin, Germany
[2] Technische Universität Berlin, Department of Food Chemistry and Toxicology, Berlin, Germany

* Correspondence: Roland Becker
E-Mail: Roland.Becker@bam.de

Number of Pages: 19
Number of Tables: 6
Number of Figures: 8

Table of content

Table S1: Overview of the analytical standards used for method development and testing material spiking experiments. 3

Figure S1 Suggested fragmentation pathway of PFOA, 6:2 FTOH and 8:2 FTCA derivatized with DMFDMA according to the mass spectrum obtained by GC-MS (EI, 70eV) 5

Figure S3 Comparison of signal intensity of DMFDMA derivatized PFAS mix (50 µg/mL) dissolved in five different solvents: **a** comparison of tert-butyl methyl ether (red); ethyl acetate (blue); acetone (yellow) and acetonitrile (green) for 16 PFAS; **b** signal intensity using methanol (grey); * 10:2 FTCA not measured; means and standard deviation (n=3) 7

Figure S4 Stability of DMFDMA derivatized products measured hourly over a 24-hour period stored at 15 °C; measured signal intensity of derivatized **a** PFCA, **b** FTOH and **c** FTCA; * 10:2 FTCA not measured . 8

DIN 32645:2008 9

Figure S5 Suggested fragmentation pathway of PFOA, 6:2 FTOH and 8:2 FTCA derivatized with PFBBr according to the mass spectrum obtained by GC-MS (EI, 70eV) 10

Figure S6 Representative mass spectra of four PFAS classes after derivatization with PFBBr: **a** PFCA: PFOA derivative with highest m/z of 594, **b** FTOH: mass spectrum of PFBBr-derivatized 6:2 FTOH, **c** FTCA: mass spectrum of derivatized 8:2 FTCA, **d** FTUCA: mass spectrum of derivatized 8:2 FTUCA 11

Figure S7 Parameter optimization of PFBBr derivatization procedure: **a** influence of incubation temperature (35 °C to 75 °C), **b** influence of the incubation time (30 min to 90 min), **c** influence of choice of carbonate (1M in H_2O), **d** influence of volume of potassium carbonate (0 µL to 20 µL) used in 1 mL aliquots of PFAS standards mixture; * 10:2 FTCA not measured; means and standard deviation (n=3) 12

Figure S8 Stability of PFBBr derivatized PFAS measured hourly over a 24-hour period stored at 15°C for PFCA and FTCA derivatives and underivatized FTOH after PFBBr treatment; * 10:2 FTCA not measured 13

Table S2 Summary of extraction efficiency for spiked cardboard material C4 at 1 µg/mL over five consecutive extraction cycles (EC). Extraction efficiency in percentage of sum of extraction values. Relative standard deviations (RSD) for each extraction efficiency, means and standard deviation (n=3) 14

Table S3 Summary of extraction efficiency for spiked agricultural fiber material F4 at 1 µg/mL over five consecutive extraction cycles (EC). Extraction efficiency in percentage of sum of extraction values. Relative standard deviations (RSD) for each extraction efficiency, means and standard deviation (n=3) 15

Table S4 Summary of extraction efficiency and recovery for spiked material C4 and F4 at 1 µg/mL in the main extraction cycles (EC1). Extraction efficiency in percentage of sum of extraction values. Recovery values in comparison to standard mix at 1 µg/mL. Relative standard deviation (RSD) for extraction efficiency and recovery of EC1, means and standard deviation (n=3) 16

Table S5 Summary of PFAS contents in paper-based FCM made from cardboard (C1-C4) and agricultural fiber (F1-F4) in the main extraction cycle (EC1) after DMFDMA derivatization. Contents in ng/g, means and standard deviations (n=3). Blind value of PFAS content in acetonitrile used for extraction. Limit of detection (LOD) and limit of quantification (LOQ) in ng/g 17

Table S6 Summary of PFAS contents in paper-based FCM made from cardboard (C1-C4) and agricultural fiber (F1-F4) in the secondary and tertiary extraction cycles (EC2; EC3) after DMFDMA derivatization. Contents and sum of contents in ng/g, means and standard deviations (n=3) 18

References 19

Table S1: Overview of the analytical standards used for method development and testing material spiking experiments.

SUBSTANCE	ABBREVIATION	SUPPLIER.	CAS #
Perfluorobutanoic acid	PFBA / C4	Merck/Sigma Aldrich (Darmstadt, Germany)	375-22-4
Perfluoropentanoic acid	PFPeA / C5	Merck/Sigma Aldrich (Darmstadt, Germany)	2706-90-3
Perfluoroheptanoic acid	PFHpA / C6	Merck/Sigma Aldrich (Darmstadt, Germany)	375-85-9
Perfluorooctanoic acid	PFOA / C8	Merck/Sigma Aldrich (Darmstadt, Germany)	335-67-1
Perfluorononanoic acid	PFNA / C9	Merck/Sigma Aldrich (Darmstadt, Germany)	375-95-1
Perfluoroundecanoic acid	PFUnDA / C11	Merck/Sigma Aldrich (Darmstadt, Germany)	2058-94-8
Perfluorotetradecanoic acid	PFTeDA / C14	Merck/Sigma Aldrich (Darmstadt, Germany)	376-06-7
1H,1H,2H,2H-Perfluorooctan-1-ol	6:2 FTOH	Merck/Sigma Aldrich (Darmstadt, Germany)	647-42-7
1H,1H,2H,2H-Perfluoro-1-decanol	8:2 FTOH	Merck/Sigma Aldrich (Darmstadt, Germany	678-39-7
Perfluorohexanoic acid	PFHxA / C7	abcr GmbH (Karlsruhe, Germany	307-24-4
Perfluorodecanoic acid	PFDA / C10	Thermo Scientific (Schwerte, Germany)	335-76-2
Perfluorododecanoic acid	PFDoDA / C12	Thermo Scientific (Schwerte, Germany)	307-55-1
Perfluorohexadecanoic acid	PFHxDA / C16	HPC Standards GmbH (Cunnersdorf, Germany)	67905-19-5
Perfluorooctadecanoic acid	PFODA / C18	HPC Standards GmbH (Cunnersdorf, Germany)	16517-11-6
2H,2H- perfluorodecanoic acid	8:2 FTCA	HPC Standards GmbH (Cunnersdorf, Germany)	27854-31-5

Table S1, continued.

2H,2H- perfluorododecanoic acid	10:2 FTCA	HPC Standards GmbH (Cunnersdorf, Germany)	53826-13-4
Internal Mass-labeled PFAS Standard Mix (MPFAC-C-ES Mass-Labelled PFAS Extraction Standards Solution (2000 ng/mL)<br>• Perfluoro-n-($^{13}C_4$)butanoic acid (MPFBA)<br>• Perfluoro-n-($^{13}C_5$)pentanoic acid (M5PFPeA)<br>• Perfluoro-n-(1,2,3,4,6-$^{13}C_5$)hexanoic acid (M5PFHxA)<br>• Perfluoro-n-(1,2,3,4-$^{13}C_4$)heptanoic acid (M4PFHpA)<br>• Perfluoro-n-($^{13}C_8$)octanoic acid (M8PFOA)<br>• Perfluoro-n-($^{13}C_9$)nonanoic acid (M9PFNA)<br>• Perfluoro-n-(1,2,3,4,5,6-$^{13}C_6$)decanoic acid (M6PFDA)<br>• Perfluoro-n-(1,2,3,4,5,6,7-$^{13}C_7$)undecanoic acid (M7PFUdA)<br>• Perfluoro-n-(1,2-$^{13}C_2$)dodecanoic acid (MPFDoA)<br>• Perfluoro-n-(1,2-$^{13}C_2$)tetradecanoic acid (M2PFTeDA)<br>• Sodium perfluoro-1-(2,3,4-$^{13}C_3$)butanesulfonate (M3PFBS)<br>• Sodium perfluoro-1-(1,2,3-$^{13}C_3$)hexanesulfonate (M3PFHxS)<br>• Sodium perfluoro-1-($^{13}C_8$)octanesulfonate (M8PFOS)		WELLINGTON LABORATORIES INC. (Ontario, Canada)	

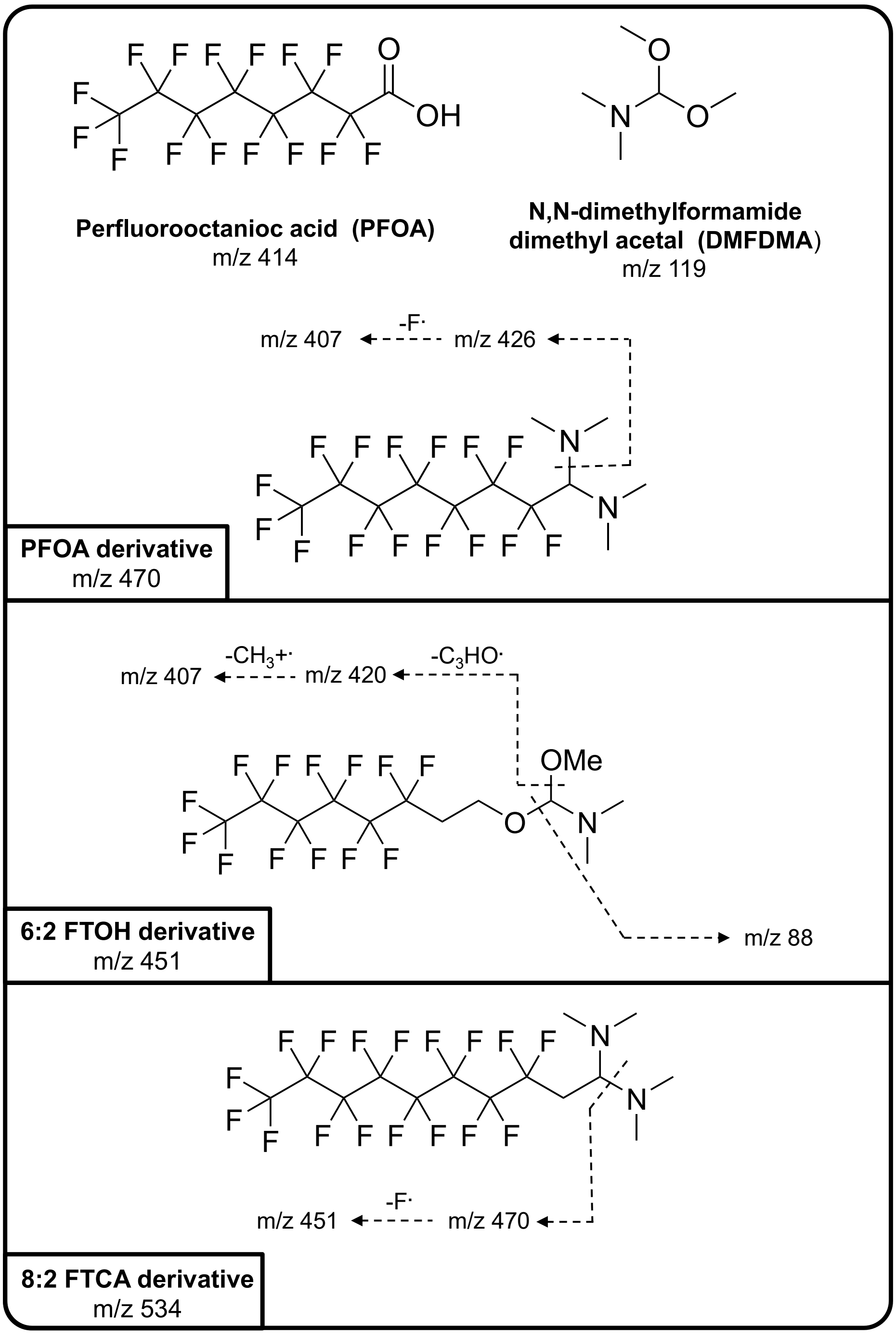

Figure S1 Suggested fragmentation pathway of PFOA, 6:2 FTOH, and 8:2 FTCA derivatized with DMFDMA according to the mass spectrum obtained by GC-MS (EI, 70eV)

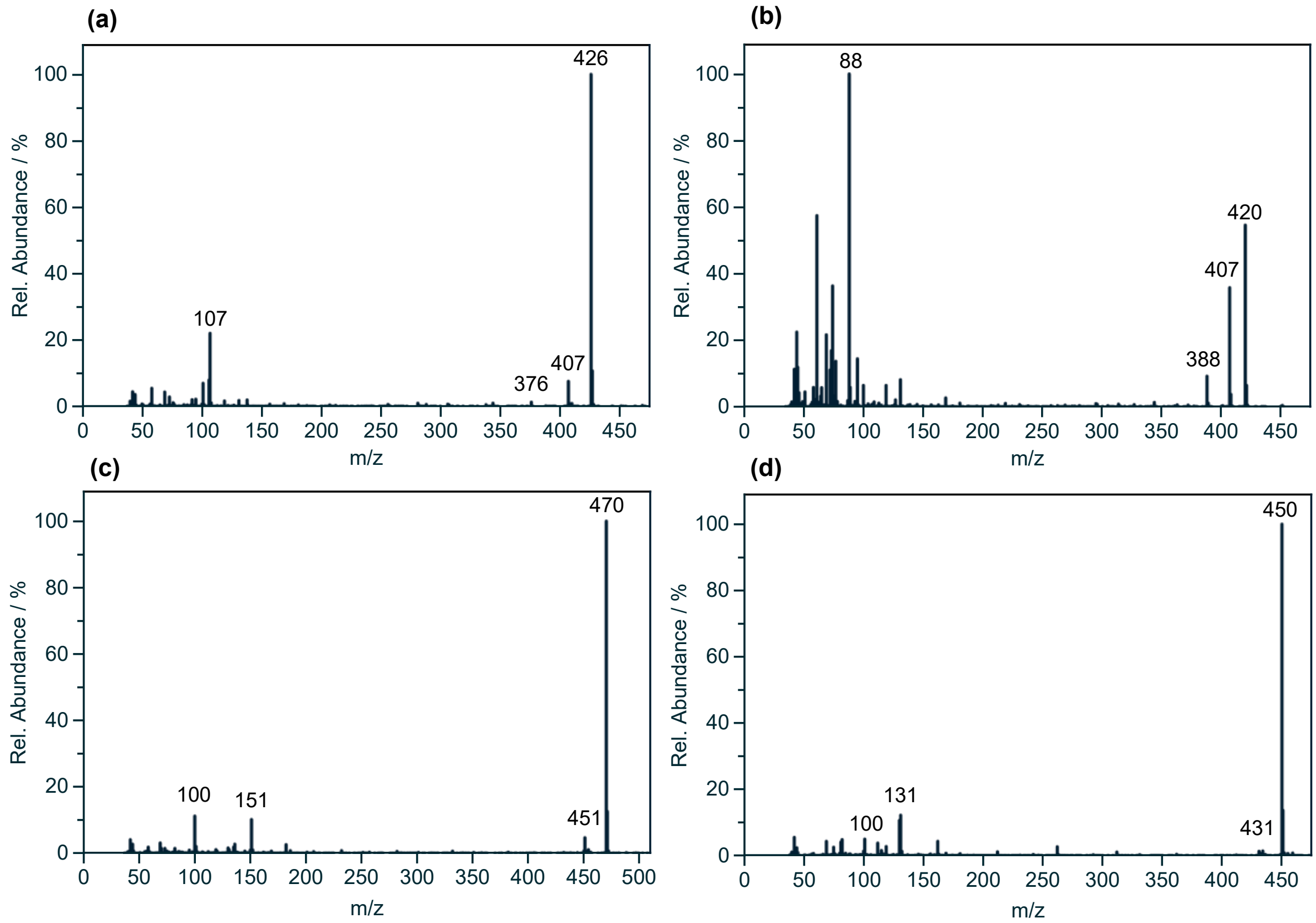

Figure S2 Representative mass spectra of four PFAS classes after derivatization with DMFDMA: **a** PFCA: PFOA derivative with highest m/z of 426, **b** FTOH: mass spectrum of DMFDMA-derivatized 6:2 FTOH, **c** FTCA: mass spectrum of derivatized 8:2 FTCA, **d** FTUCA: mass spectrum of derivatized 8:2 FTUCA

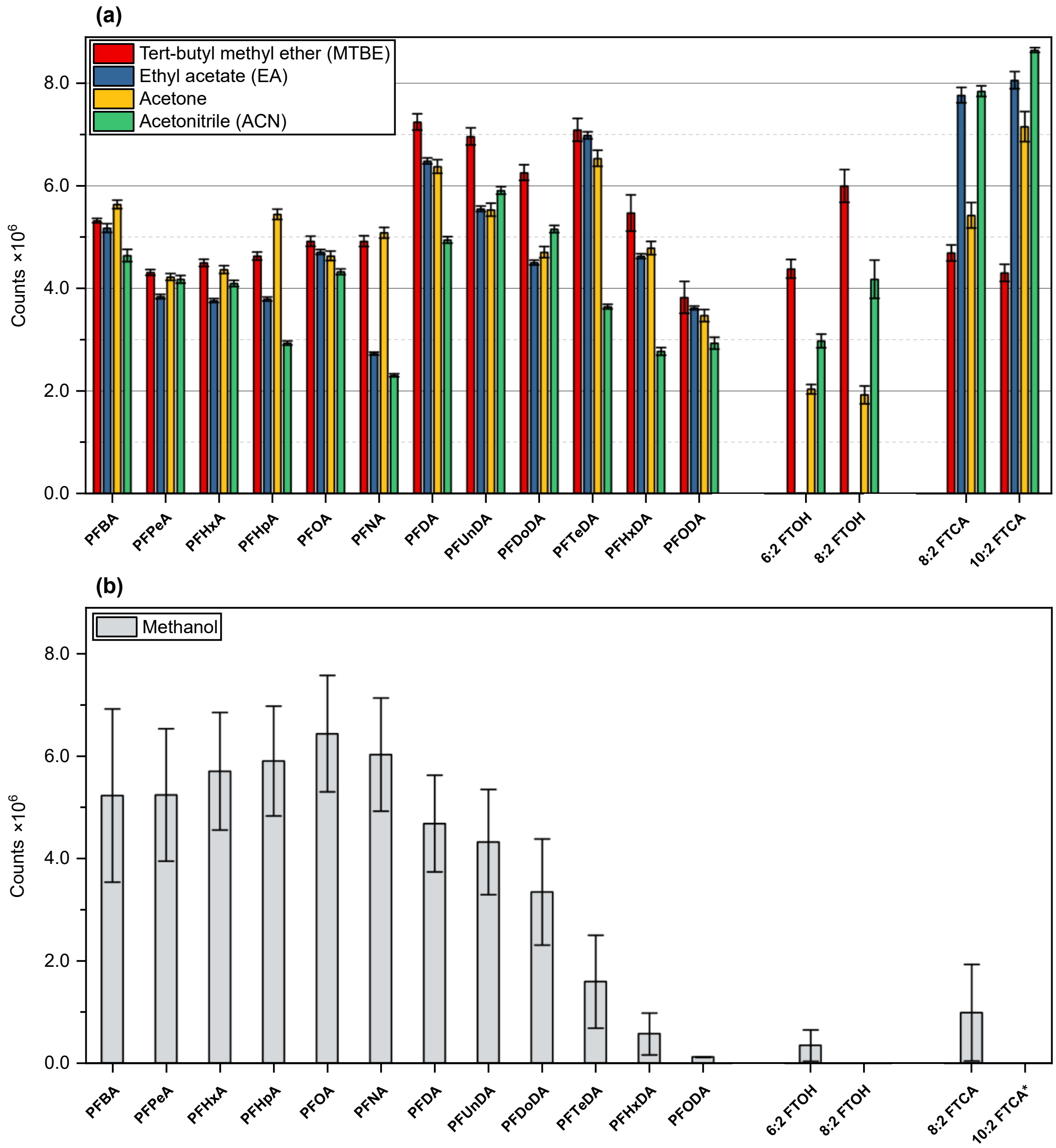

Figure S3 Comparison of signal intensity of DMFDMA derivatized PFAS mix (50 µg/mL) dissolved in five different solvents: **a** comparison of tert-butyl methyl ether (red); ethyl acetate (blue); acetone (yellow) and acetonitrile (green) for 16 PFAS; **b** signal intensity using methanol (grey); * 10:2 FTCA not measured; means and standard deviation (n=3)

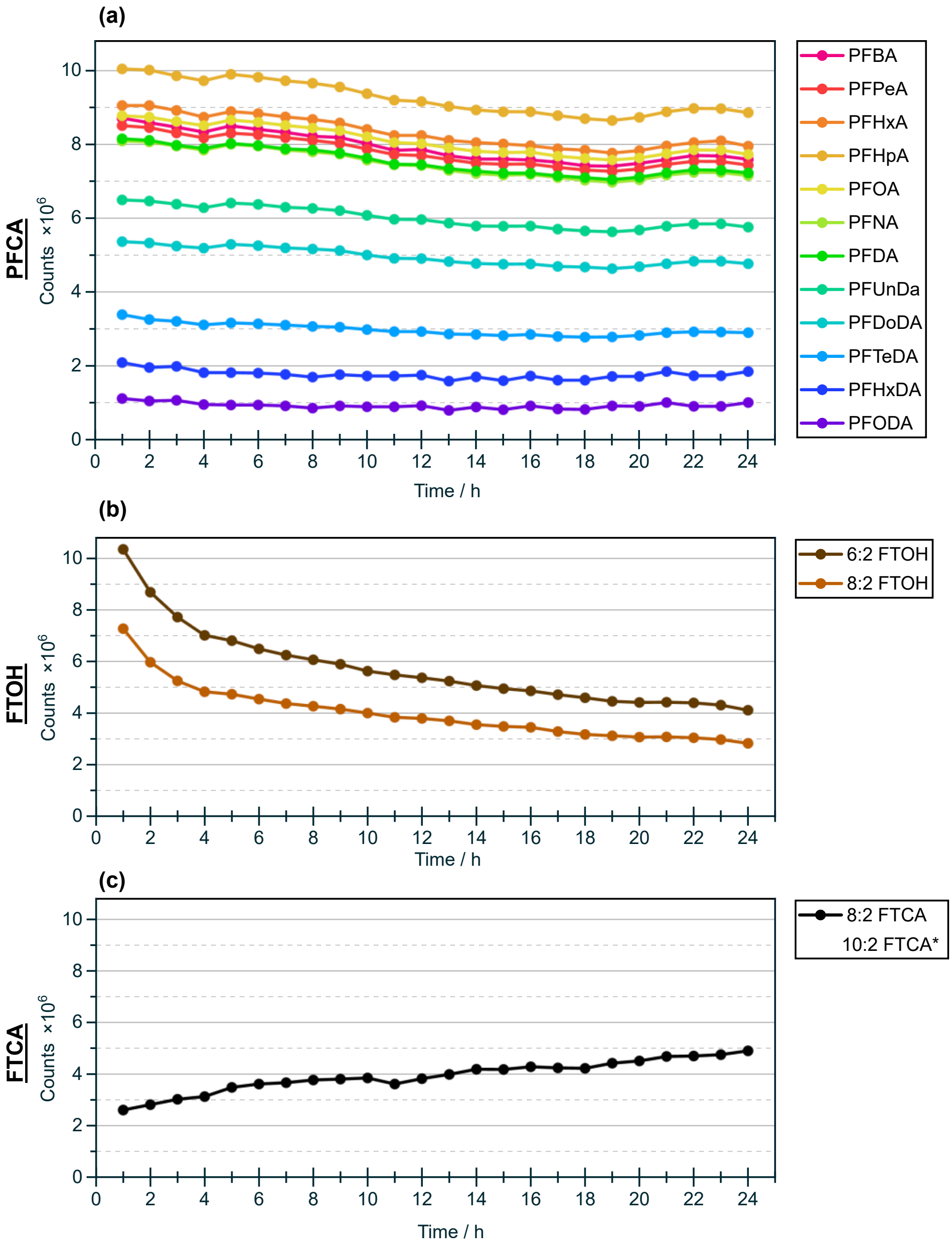

Figure S4 Stability of DMFDMA derivatized products measured hourly over a 24-hour period stored at 15 °C; measured signal intensity of derivatized **a** PFCA, **b** FTOH and **c** FTCA; * 10:2 FTCA not measured

DIN 32645:2008

The determination of the detection limit (LOD) and the quantification limit (LOQ) was performed by using the calibration curve method based on DIN 32645:2008.

$$LOD = s_{x;0} \times t_{f;\alpha} \times \sqrt{\frac{1}{m} + \frac{1}{n} + \frac{\overline{\mathrm{x}}}{Q_x}} \qquad (SI - 1)$$

$$LOQ = k \times s_{x;0} \times t_{f;\frac{\alpha}{2}} \times \sqrt{\frac{1}{m} + \frac{1}{n} + \frac{[(3 \times LOD) - \overline{\mathrm{x}}]^2}{Q_x}} \qquad (SI - 2)$$

with $s_{x;0} \approx \frac{s_{y,x}}{b}$ the standard deviation of the procedure ($s_{y,x}$ = standard deviation of the residuals of the calibration samples and b = slope of the calibration curve); $t_{f;\mathrm{a}}$ = quantile of the t-distribution for a one-sided test and $t_{f;\frac{\alpha}{2}}$ = quantile of the t-distribution for a two-sided test with $f = n-2$ degrees of freedom; n = number of calibration samples; m = number of measurements on the calibration sample; $\overline{\mathrm{x}}$ = arithmetic mean of the mass concentration of the calibration samples; Q_x = sum of squared differences of the individual calibration levels from the mean calibration level; 1/k = relative uncertainty of the result used to characterize the detection limit (33.3 % at k = 3) [1].

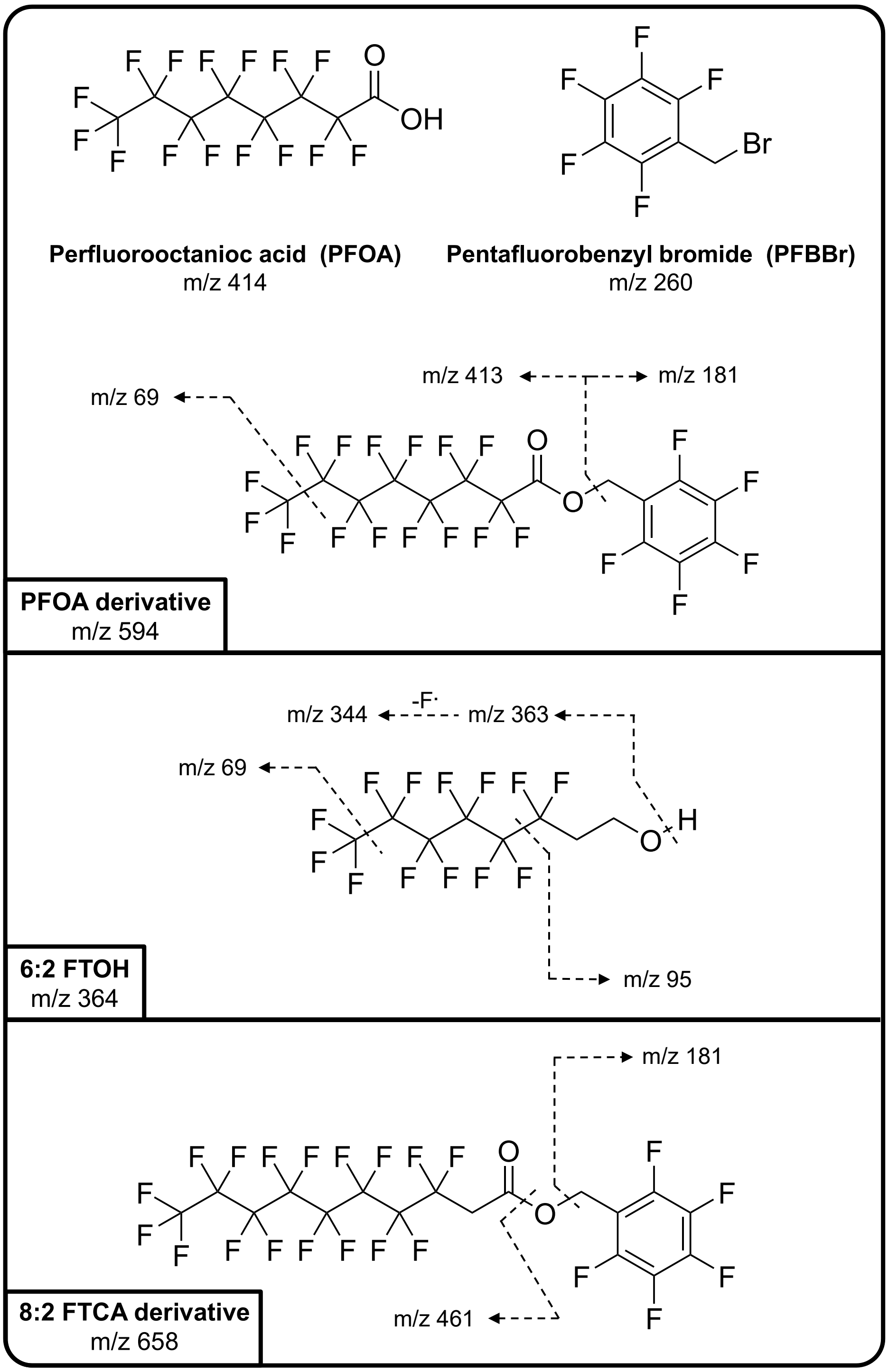

Figure S5 Suggested fragmentation pathway of PFOA, 6:2 FTOH, and 8:2 FTCA derivatized with PFBBr according to the mass spectrum obtained by GC-MS (EI, 70eV)

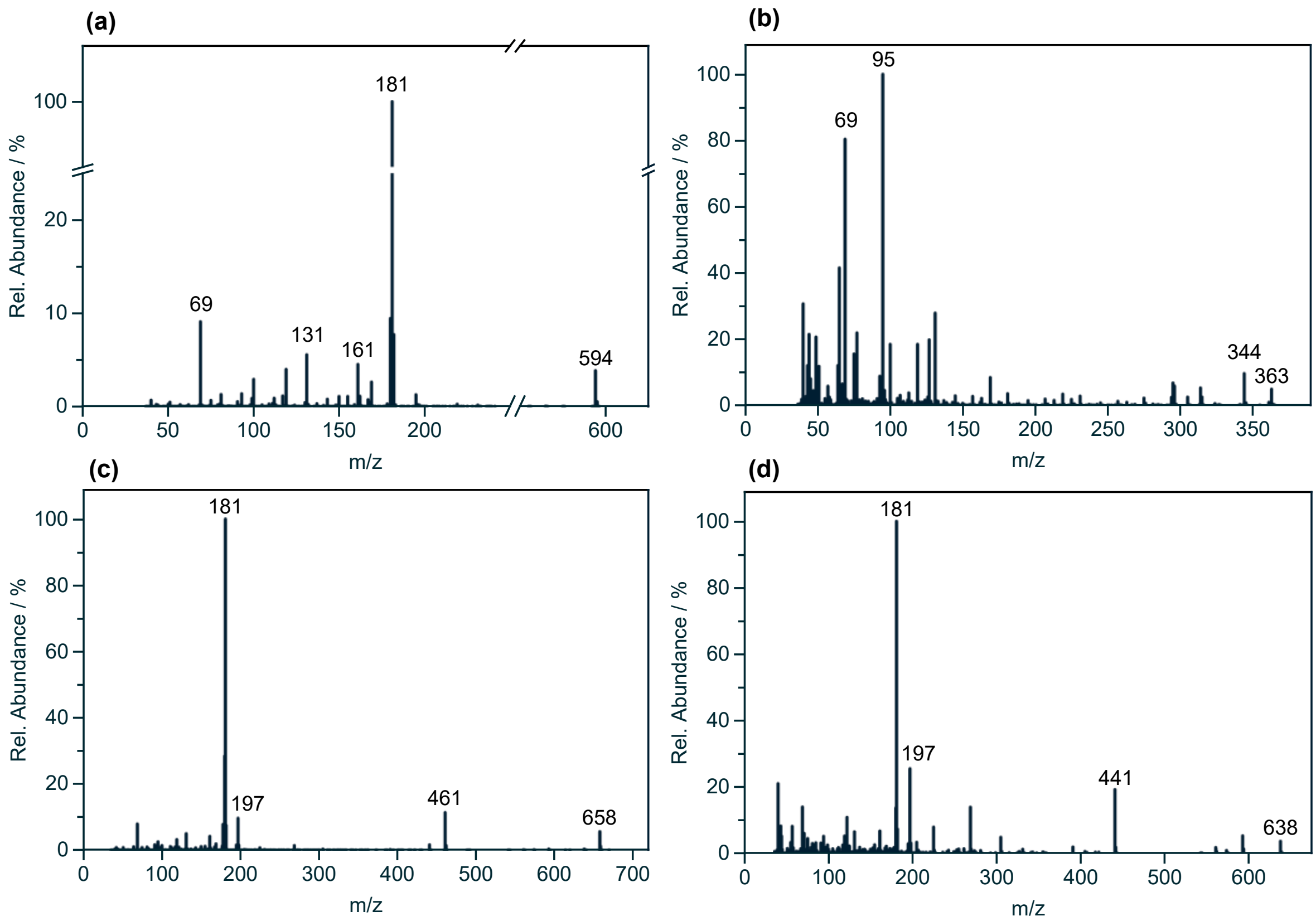

Figure S6 Representative mass spectra of four PFAS classes after derivatization with PFBBr: **a** PFCA: PFOA derivative with highest m/z of 594, **b** FTOH: mass spectrum of PFBBr-derivatized 6:2 FTOH, **c** FTCA: mass spectrum of derivatized 8:2 FTCA, **d** FTUCA: mass spectrum of derivatized 8:2 FTUCA

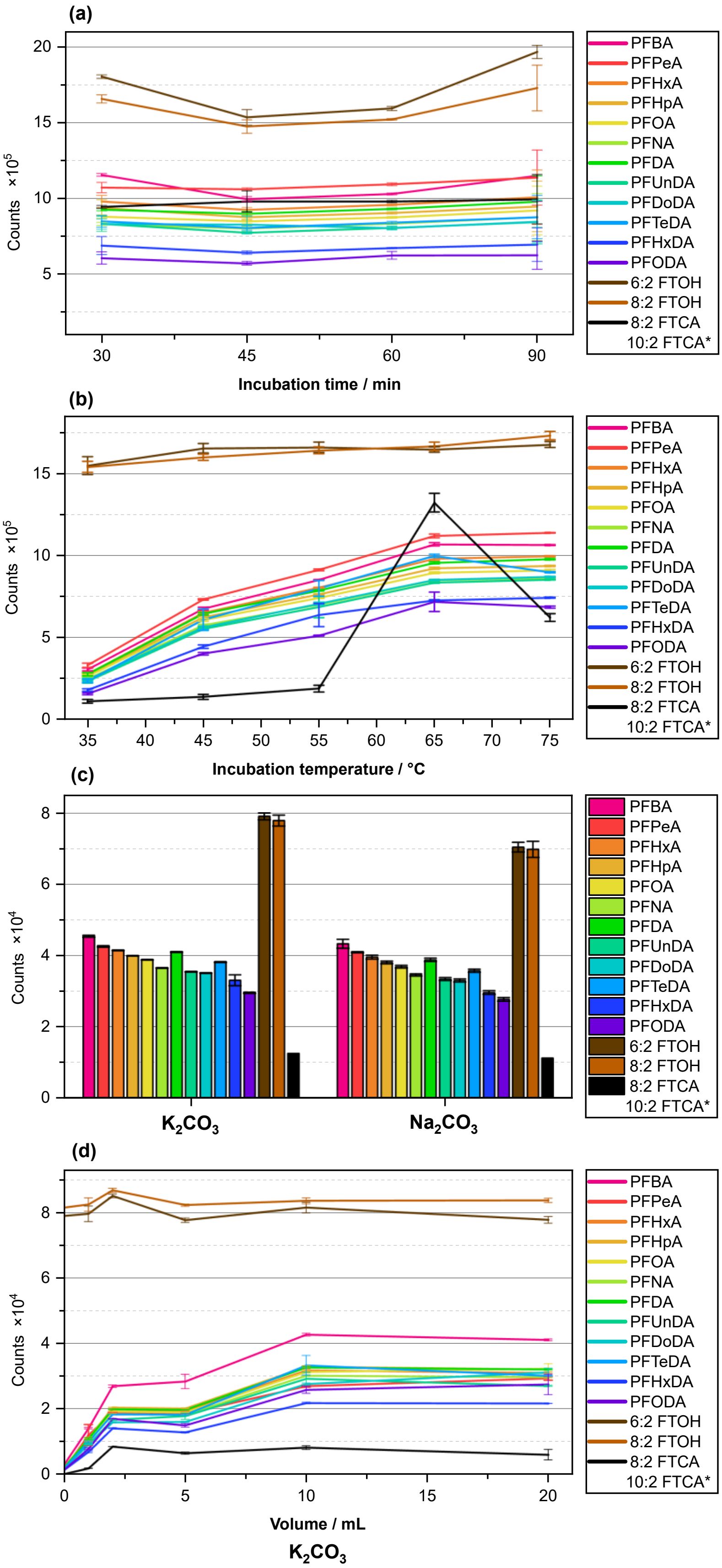

Figure S7 Parameter optimization of PFBBr derivatization procedure: **a** influence of incubation temperature (35 °C to 75 °C), **b** influence of the incubation time (30 min to 90 min), **c** influence of choice of carbonate (1M in H_2O), **d** influence of volume of potassium carbonate (0 µL to 20 µL) used in 1 mL aliquots of PFAS standards mixture; * 10:2 FTCA not measured; means and standard deviation (n=3)

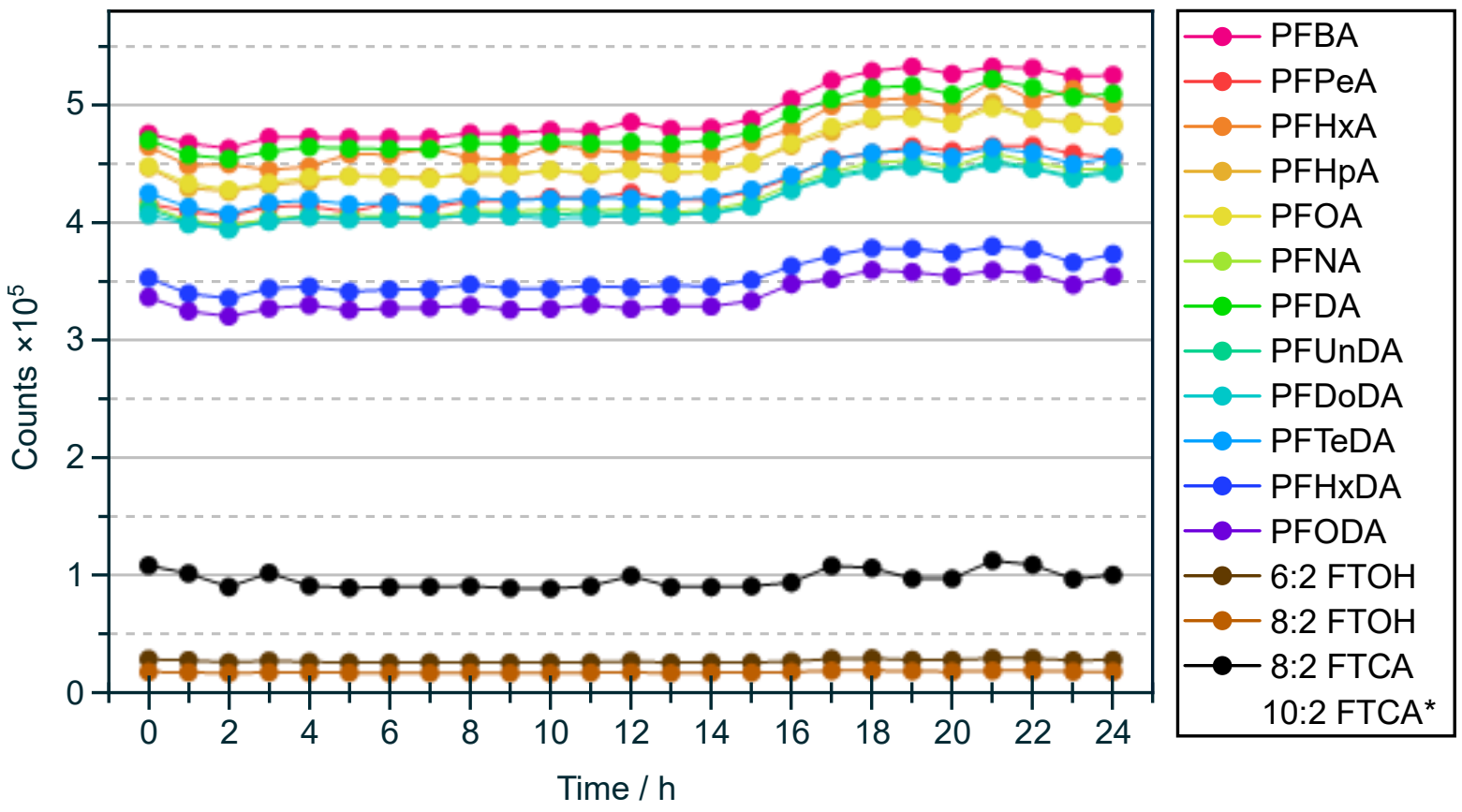

Figure S8 Stability of PFBBr derivatized PFAS measured hourly over a 24-hour period stored at 15°C for PFCA and FTCA derivatives and underivatized FTOH after PFBBr treatment; * 10:2 FTCA not measured

Table S2 Summary of extraction efficiency for spiked cardboard material C4 at 1 µg/mL over five consecutive extraction cycles (EC). Extraction efficiency in percentage of sum of extraction values. Relative standard deviations (RSD) for each extraction efficiency, means and standard deviation (n=3)

**Cardboard material (C4) spiked at 1µg/mL (n=3)**										
	**Extraction efficiency of EC1 /%**	**RSD / %**	**Extraction efficiency of EC2 /%**	**RSD / %**	**Extraction efficiency of EC3 /%**	**RSD / %**	**Extraction efficiency of EC4 /%**	**RSD / %**	**Extraction efficiency of EC5 /%**	**RSD / %**
PFBA	69.91	1.06	17.22	1.24	6.60	0.45	3.27	0.44	3.00	0.20
PFPeA	73.78	1.00	16.55	1.14	5.20	0.21	2.30	0.35	2.16	0.22
PFHxA	76.29	1.04	16.12	1.09	4.49	0.18	1.60	0.26	1.49	0.21
PFHpA	76.37	0.66	16.43	1.55	3.31	2.22	2.01	0.22	1.88	0.26
PFOA	76.87	0.82	16.09	1.19	4.13	0.30	1.52	0.23	1.38	0.18
PFNA	75.33	2.33	16.06	0.91	5.56	1.56	1.58	0.26	1.46	0.17
PFDA	77.54	0.48	16.46	1.54	3.03	1.99	1.57	0.18	1.40	0.17
PFUnDA	76.54	1.19	16.38	1.26	4.34	0.14	1.51	0.16	1.24	0.12
PFDoDA	74.99	1.75	16.83	1.48	5.06	0.43	1.79	0.14	1.34	0.15
PFTeDA	65.00	3.89	19.26	2.43	9.25	1.89	3.87	0.16	2.63	0.59
PFHxDA	50.80	5.08	21.80	2.89	15.56	3.78	7.08	0.28	4.77	1.40
PFODA	40.48	3.99	24.94	3.77	18.97	1.51	9.24	1.07	6.37	1.55
6:2 FTOH	100	0	-	-	-	-	-	-	-	-
8:2 FTOH	100	47.14	-	-	-	-	-	-	-	-
8:2 FTCA	44.70	3.47	28.07	0.50	18.67	2.87	5.55	0.82	3.01	0.21
10:2 FTCA	79.06	1.68	10.86	1.25	6.68	2.56	2.67	0.93	0.73	0.60

–: not detected

Table S3 Summary of extraction efficiency for spiked agricultural fiber material F4 at 1 µg/mL over five consecutive extraction cycles (EC). Extraction efficiency in percentage of sum of extraction values. Relative standard deviations (RSD) for each extraction efficiency, means and standard deviation (n=3)

**Agricultural fiber material (F4) spiked at 1µg/mL (n=3)**										
	**Extraction efficiency of EC1 / %**	**RSD / %**	**Extraction efficiency of EC2 / %**	**RSD / %**	**Extraction efficiency of EC3 / %**	**RSD / %**	**Extraction efficiency of EC4 / %**	**RSD / %**	**Extraction efficiency of EC5 / %**	**RSD / %**
PFBA	59.44	2.42	22.08	1.20	9.96	0.66	5.41	0.46	3.10	0.26
PFPeA	61.26	2.71	21.86	1.31	9.51	0.75	4.81	0.45	2.56	0.35
PFHxA	59.82	2.94	22.49	1.35	9.85	0.76	5.09	0.57	2.74	0.43
PFHpA	56.76	3.06	23.28	1.19	11.13	0.93	5.72	0.66	3.10	0.47
PFOA	52.33	2.97	23.79	0.97	12.54	1.00	7.15	0.75	4.18	0.57
PFNA	49.38	3.22	23.96	1.09	13.57	1.04	8.12	0.80	4.98	0.67
PFDA	45.58	3.16	24.38	1.02	14.61	1.13	9.40	1.01	6.02	0.63
PFUnDA	41.47	2.81	24.61	0.84	15.93	1.29	11.03	1.04	6.96	0.60
PFDoDA	38.69	3.05	25.02	1.20	16.78	1.44	11.64	0.99	7.88	0.54
PFTeDA	33.23	2.73	25.86	1.04	18.00	1.53	13.57	1.26	9.34	0.52
PFHxDA	35.39	3.57	25.48	1.38	17.24	1.75	12.78	1.47	9.11	0.51
PFODA	37.52	4.89	26.09	1.05	15.51	2.25	12.32	1.72	8.56	0.93
6:2 FTOH	90.79	0.66	7.76	0.57	1.45	0.10	0.00	0.00	0.00	0.00
8:2 FTOH	90.04	1.19	8.58	1.02	1.37	0.20	0.00	0.00	0.00	0.00
8:2 FTCA	74.63	3.38	15.08	3.00	5.54	0.10	3.09	0.17	1.66	0.45
10:2 FTCA	72.56	2.88	15.05	2.06	7.50	0.41	3.47	0.53	1.43	0.36

Table S4 Summary of extraction efficiency and recovery for spiked material C4 and F4 at 1 µg/mL in the main extraction cycles (EC1). Extraction efficiency in percentage of sum of extraction values. Recovery values in comparison to standard mix at 1 µg/mL. Relative standard deviation (RSD) for extraction efficiency and recovery of EC1, means and standard deviation (n=3)

Spiking at 1 µg/mL (n=3)										
	Cardboard material C4					Agricultural fiber material F4				
	Extraction efficiency of EC1 / %	RSD / %	Recovery of EC1 / %	RSD / %	Recovery / %	Extraction efficiency of EC1 / %	RSD / %	Recovery of EC1 / %	RSD / %	Recovery / %
PFBA	69.91	1.06	80.33	2.33	107.69	59.44	2.42	44.4	3.53	68.4
PFPeA	73.78	1.00	84.01	1.71	108.78	61.26	2.71	48.2	4.36	72.9
PFHxA	76.29	1.04	84.25	1.72	107.02	59.82	2.94	47.4	4.52	73.1
PFHpA	76.37	0.66	83.93	2.13	105.62	56.76	3.06	43.2	4.44	69.4
PFOA	76.87	0.82	82.72	2.31	104.49	52.33	2.97	37.5	3.99	63.5
PFNA	75.33	2.33	81.70	2.52	105.14	49.38	3.22	31.8	3.96	55.9
PFDA	77.54	0.48	79.49	2.41	99.47	45.58	3.16	26.5	3.48	49.1
PFUnDA	76.54	1.19	76.29	2.79	96.95	41.47	2.81	20.1	2.68	39.7
PFDoDA	74.99	1.75	71.84	3.85	92.82	38.69	3.05	17.0	2.58	35.5
PFTeDA	65.00	3.89	55.98	6.53	80.53	33.23	2.73	11.5	1.59	26.6
PFHxDA	50.80	5.08	42.41	7.04	73.59	35.39	3.57	14.7	2.38	32.5
PFODA	40.48	3.99	41.57	8.52	86.67	37.52	4.89	21.6	4.55	45.6
6:2 FTOH	100	0	17.4	14.26	17.4	90.79	0.66	118.9	8.43	130.9
8:2 FTOH	100	47.14	10.6	4.01	10.6	90.04	1.19	129.8	8.81	144.2
8:2 FTCA	44.70	3.47	10.4	0.19	21.3	74.63	3.38	27.8	3.58	35.4
10:2 FTCA	79.06	1.68	34.6	0.26	42.3	72.56	2.88	18.2	0.94	23.9

Table S5 Summary of PFAS contents in paper-based FCM made from cardboard (C1-C4) and agricultural fiber (F1-F4) in the main extraction cycle (EC1) after DMFDMA derivatization. Contents in ng/g, means and standard deviations (n=3). Blind value of PFAS content in acetonitrile used for extraction. Limit of detection (LOD) in ng/g

			**Main Extraction (EC1)**							
	**Solvent Blank / ng/mL**	**LOD / ng/g**	**Cardboard / ng/g**				**Agricultural Fiber / ng/g**			
			**C1**	**C2**	**C3**	**C4**	**F1**	**F2**	**F3**	**F4**
PFBA	< LOD: 0.92	7.14	-	-	-	62.59 ± 3.91	-	-	-	-
PFPeA	1.52	5.11	-	-	-	33.54 ± 5.04	-	-	-	-
PFHxA	< LOD	5.81	-	-	-	31.12 ± 2.27	24.25 ± 0.94	-	27.20 ± 0.51	-
PFHpA	2.66	6.79	-	-	-	35.88 ± 4.90	-	-	27.71 ± 0.44	-
PFOA	3.35	6.00	-	-	-	-	-	-	70.01 ± 1.13	-
PFNA	< LOD	4.24	-	-	-	-	-	-	26.66 ± 0.69	-
PFDA	1.94	5.42	-	-	-	-		-	141.71 ± 4.12	-
PFUnDA	< LOD: 0.65	6.12	-	-	-	-	-	-	19.17 ± 0.57	-
PFDoDA	< LOD	22.45	-	-	-	-	-	-	55.27 ± 5.04	-
PFTeDA	< LOD	28.29	-	-	-	-	-	-	-	-
PFHxDA	< LOD	28.54	-	-	-	-	-	-	-	-
PFODA	< LOD	17.60	-	-	-	-	-	-	-	-
6:2 FTOH	< LOD	48.65	-	-	-	-	-	-	-	-
8:2 FTOH	< LOD	34.70	-	-	-	-	-	-	-	-
8:2 FTCA	< LOD	45.35	-	-	-	-	-	-	162.63 ± 1.67	-
10:2 FTCA	< LOD	45.94	-	-	-	-	-	-	-	-

< LOD: concentrations below limit of detection value.

–: not detected.

Table S6 Summary of PFAS contents in paper-based FCM made from cardboard (C1-C4) and agricultural fiber (F1-F4) in the secondary and tertiary extraction cycles (EC2; EC3) after DMFDMA derivatization. Contents and sum of contents in ng/g, means and standard deviations (n=3)

	**Secondary Extraction (EC2)**								**Tertiary Extraction (EC3)**								**Sum of PFAS content**		
	**Cardboard / ng/g**				**Agricultural Fiber / ng/g**				**Cardboard / ng/g**				**Agricultural Fiber / ng/g**				**/ ng/g**		
	**C1**	**C2**	**C3**	**C4**	**F1**	**F2**	**F3**	**F4**	**C1**	**C2**	**C3**	**C4**	**F1**	**F2**	**F3**	**F4**	**C4**	**F1**	**F3**
PFBA	-	-	-	25.31 ± 1.75	-	-	-	-	-	-	-	< LOD: 12.99 ± 1.78	-	-	-	-	100.89	-	-
PFPeA	-	-	-	11.56 ± 1.44	-	-	-	-	-	-	-	< LOD: 3.83 ± 0.34	-	-	-	-	48.93	-	-
PFHxA	-	-	-	19.44 ± 2.24	13.44 ± 0.88	-	16.32 ± 1.56	-	-	-	-	-	-	-	-	-	50.56	37.69	43.52
PFHpA	-	-	-	22.80 ± 2.19	-	-	17.95 ± 0.79	-	-	-	-	-	-	-	-	-	58.68	-	45.66
PFOA	-	-	-	-	-	-	26.56 ± 5.10	-	-	-	-	-	-	-	15.69 ± 9.25	-	-	-	112.26
PFNA	-	-	-	-	-	-	-	-	-	-	-	-	-	-	-	-	-	-	26.66
PFDA	-	-	-	-	-	-	65.23 ± 7.91	-	-	-	-	-	-	-	54.44 ± 16,97	-	-	-	261.38
PFUnDA	-	-	-	-	-	-	< LOD: 11.23 ± 0.72	-	-	-	-	-	-	-	-	-	-	-	30.94
PFDoDA	-	-	-	-	-	-	< LOD: 7.95 ± 4.50	-	-	-	-	-	-	-	-	-	-	-	63.22
PFTeDA	-	-	-	-	-	-	-	-	-	-	-	-	-	-	-	-	-	-	-
PFHxDA	-	-	-	-	-	-	-	-	-	-	-	-	-	-	-	-	-	-	-
PFODA	-	-	-	-	-	-	-	-	-	-	-	-	-	-	-	-	-	-	-
6:2 FTOH	-	-	-	-	-	-	-	-	-	-	-	-	-	-	-	-	-	-	-
8:2 FTOH	-	-	-	-	-	-	-	-	-	-	-	-	-	-	-	-	-	-	-
8:2 FTCA	-	-	-	-		-	112.29 ± 8.25	-	-	-	-	-		-	-	-	-		274.92
10:2 FTCA	-	-	-	-	-	-	-	-	-	-	-	-	-	-	-	-	-	-	-

< LOD: concentrations below limit of detection value.
–: not detected.

References

1. Deutsches Institut für Normung e.V. DIN 32645:2008-11; Chemische Analytik – Nachweis-, Erfassungs- und Bestimmungsgrenze unter Wiederholbedingungen – Begriffe, Verfahren, Auswertung. 28 (2008).
